# *Dickeya zeae* strains isolated from rice, banana and clivia rot plants show great virulence differentials

**DOI:** 10.1186/s12866-018-1300-y

**Published:** 2018-10-18

**Authors:** Ming Hu, Jieling Li, Ruiting Chen, Wenjun Li, Luwen Feng, Lei Shi, Yang Xue, Xiaoyin Feng, Lianhui Zhang, Jianuan Zhou

**Affiliations:** 1 Integrative Microbiology Research Centre, South China Agricultural University, Guangzhou, 510642 China; 2 Guangdong Province Key Laboratory of Microbial Signals and Disease Control, Department of Plant Pathology, South China Agricultural University, Guangzhou, 510642 China

**Keywords:** *Dickeya zeae*, Virulence factor, Pathogenicity, Host range

## Abstract

**Background:**

*Dickeya zeae* is the causal agent of maize and rice foot rot diseases, but recently it was also found to infect banana and cause severe losses in China. Strains from different sources showed significant diversity in nature, implying complicated evolution history and pathogenic mechanisms.

**Results:**

*D. zeae* strains were isolated from soft rot banana plants and ornamental monocotyledonous *Clivia miniata*. Compared with *D. zeae* strain EC1 isolated from rice, clivia isolates did not show any antimicrobial activity, produced less extracellular enzymes, had a much narrow host ranges, but released higher amount of extracellular polysaccharides (EPS). In contrast, the banana isolates in general produced more extracellular enzymes and EPS than strain EC1. Furthermore, we provided evidence that the banana *D. zeae* isolate MS2 produces a new antibiotic/phytotoxin(s), which differs from the zeamine toxins produced by rice pathogen *D. zeae* strain EC1 genetically and in its antimicrobial potency.

**Conclusions:**

The findings from this study expanded the natural host range of *D. zeae* and highlighted the genetic and phenotypic divergence of *D. zeae* strains. Conclusions can be drawn from a series of tests that at least two types of *D. zeae* strains could cause the soft rot disease of banana, with one producing antimicrobial compound while the other producing none, and the *D. zeae* clivia strains could only infect monocot hosts. *D. zeae* strains isolated from different sources have diverse virulence characteristics.

**Electronic supplementary material:**

The online version of this article (10.1186/s12866-018-1300-y) contains supplementary material, which is available to authorized users.

## Background

*Dickeya* species (spp.), is one of the top ten important bacterial phytopathogens in the world, and has been listed as a plant quarantine pest in China since 2007 [1, 2]. There are currently 8 species in this genus, including *D. dianthicola*, *D. dadantii*, *D. zeae*, *D. chrysanthemi*, *D. paradisiaca*, *D. solani*, *D. aquatic* and *D. fangzhongdai* [3–6], and among them, *D. dadantii*, *D. zeae* and *D. solani* usually cause devastating disease, resulting in a considerable loss in crop yield, especially on potato, rice and banana [7–12].

Being located in a divergent evolutionary branch, *D. zeae* bacteria were reported to infect a wide range of plants all over the world, including 4 kinds of natural dicotyledonous hosts such as potato, tobacco, *Chrysanthemum* and *Philodendron*, and 6 kinds of natural monocotyledonous hosts such as maize, rice, banana, pineapple, *Brachiaria* and hyacinth (Additional file 1) [3, 8, 10, 12–32], besides, other 32 kinds of plants were reported as artificial hosts of *D. zeae* (Additional file 1) [13, 33, 34].

Latent infection appears to be a common trait of *D*. *zeae*. For instance, rice foot rot disease occurred severely in Jiangsu Province in 1980s resulting in about 90% losses on rice yield [35], but inactive for about 10 years till in 2000s, outbreak occurred in Fujian, Hunan, Guizhou, and Shandong provinces with different disease incidences ranging from 15 to 100% [36–38]. The disease currently occurs in Guangdong Province occasionally and sporadically. However, banana soft rot disease also caused by *D. zeae* has become a severe problem in Guangdong Province since 2009, with over 6000 ha of banana plantation being infected from 2010 to 2012 [10, 12]. The disease is now spreading to the major banana plantation fields in China in Provinces of Fujian, Yunnan, Hainan and Guangxi. Study on *D. zeae* banana strain is rare and its pathogenicity mechanisms are unclear.

Among the *Dickeya* spp., *D. dadantii* is perhaps the most characterized representative. The pathogen produces a range of virulence factors including cell wall degrading enzymes, type III secretion system (T3SS), siderphores, and indigoidine pigment, which collectively contribute to bacterial virulence [39–42]. Different from other *Dickeya* species, *D. zeae* can infect both dicots and monocots [7], indicating the existence of additional virulence factors. Genetic analysis and genome sequence comparison identified a *zms* gene cluster in *D. zeae* rice strains, which encodes the biosynthesis of zeamine phytotoxins capable of inhibiting rice seeds germination and growth [11, 43, 44]. Characterization of the *D. zeae* rice isolate EC1 also unveiled a quorum sensing (QS) system that produces and senses acyl homoserine lactone (AHL) signal to regulate expression of virulence associated genes, as well as a MarR family transcriptional regulator SlyA, and hereafter, to influence cell motility and biofilm formation [7, 44]. In addition, strain EC1 also relies on a Fis transcriptional regulator to directly regulate the expression of *zms* genes and production of cell wall degrading enzymes [45]. However, many detailed virulence regulatory mechanisms of *D. zeae* still remain unknown, especially in the banana strains, and few virulence factors related to host specificity have been determined.

In this study, *D. zeae* strains from rice, banana and monocot ornamental clivia plants that respectively cause severe bacterial rot disease in fields were collected to explore their phylogenetic relationship, and virulence differentials among strains were investigated by comparing the production of major virulence factors including extracellular enzymes, extracellular polysaccharides (EPS) and phytotoxins, nematode-killing activity and pathogenicity on many reported host plants. The findings from this study may present a new insight and clues for the control of bacterial soft rot diseases on crops.

## Results

### Strains isolated from soft rot banana and clivia plants were classified as *D. zeae* based on phylogenetic analysis

**Fig. 1:**
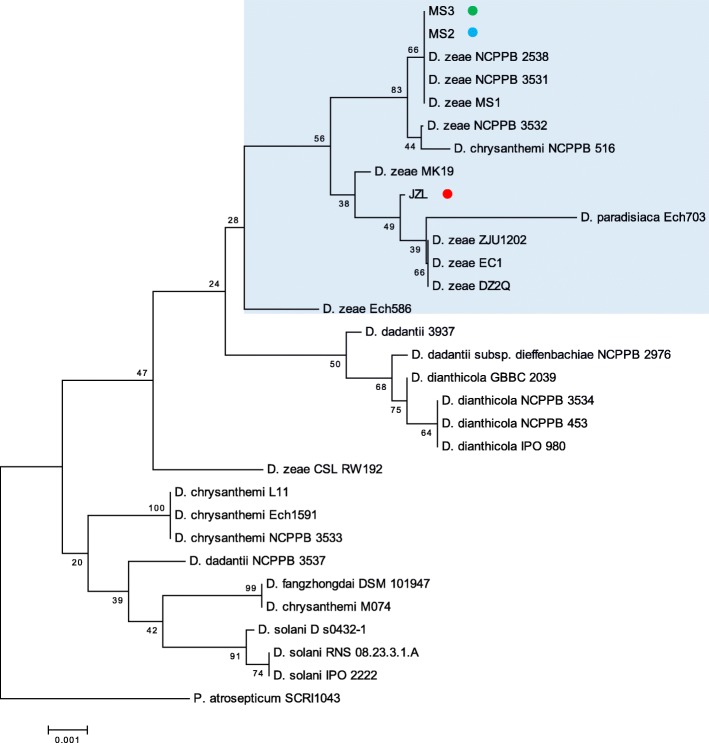
Phylogenetic tree based on the *16S rRNA* sequences of *Dickeya* species. Consensus sequences were aligned with ClustalW and trimmed in size of 654 bp. Bootstrap value after 1000 replicates is expressed as percentages. *Pectobacterium atrosepticum* SCRI1043 is included as an outgroup. Bar, 0.1% substitution rate per site

**Fig. 2:**
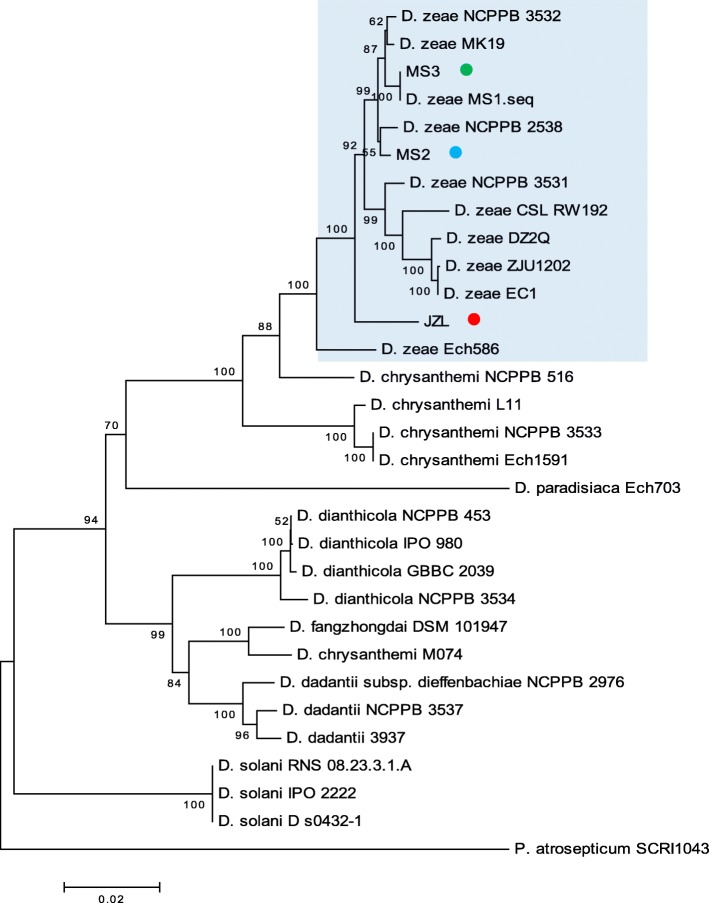
Joint phylogenetic tree based on the concatenated nucleotide sequences of *atpD*, *gyrB*, *infB* and *rpoB* of *Dickeya* strains*.* Consensus sequences were aligned with ClustalW and trimmed in the following sizes: *atpD*, 642 bp; *gyrB*, 745 bp; *infB*, 1042 bp; *rpoB*, 1000 bp. All the sequences of a same strain were assembled for constructing the joint Neighbor-joining tree. Bootstrap values after 1000 replicates are expressed as percentages. *P. atrosepticum* SCRI1043 was included as an outgroup. Bar, 2% substitution rate per site

*D. zeae* was reported to be the causal agent of banana soft rot disease in Guangzhou, China [10, 12]. In this study, strains MS2 and MS3 that caused severe soft rot disease were respectively isolated from the basal pseudostems of banana plants in Nansha and Panyu Districts in Guangzhou city in 2012; and strains JZL1, JZL2 and JZL7 were isolated from the decayed centre leaves of clivia plants collected at Fangcun flower market in Guangzhou in 2017. The pure cultures of these isolates were inoculated to the corresponding banana and clivia plants, and typical soft rot symptoms were noticed (data not shown), validating their roles as banana and clivia pathogens, respectively. To identify the taxonomic status of these pathogens, MLSA analysis was performed based on the partial sequences of *16S rRNA* gene, and the other four housekeeping genes including *atpD*, *gyrB*, *infB* and *rpoB.* Results showed that the three JZL strains contain identical housekeeping gene sequences, thus were designated as strain JZL. The alignment results showed that strains JZL, MS2 and MS3 clustered in the same branch with known *D. zeae* strains both in the *16S rDNA* tree (Fig. 1) and the joint phylogenetic tree built on the concatenated nucleotide sequences of *atpD*, *gyrB*, *infB* and *rpoB* from 30 *Dickeya* species and strains (Fig. 2)*.*

The *16S rDNA* sequences of strains MS2 and MS3 are fully identical to those of *D. zeae* MS1 (from banana) [12], 99% identical to those of *D. zeae* rice isolates EC1 [11, 43], ZJU1202 [19] and DZ2Q [20]. Strains MS2 and MS3 had the identical *atpD* and *gyrB* sequences, showing 99% identity to their counterparts of strain MS1. In contrast, while strains MS3 and MS1 contain the same *infB* and *rpoB* gene sequences, strain MS2 shared 99% identity to those of strain MS1. Cumulatively, these data indicate that strains MS2 and MS3 are members of *D. zeae*, closely related to the previously identified *D. zeae* strain MS1 [12].

The *16S rDNA* sequence of strain JZL is 99% identical to those of *D. zeae* strains EC1, Ech586 (from *Philodendron*) and MK19 (from river water), and 98% identical to that of strain MS2. The assembled sequence of *atpD*, *gyrB*, *infB* and *rpoB* (3429 bp) of MS2 is 99% identical to those of strains MS1 and MS3, 98% identical to that of strain EC1, and 97% identical to that of strain Ech586; and the assembled sequence of strain JZL is 97% identical to those of strains EC1 and Ech586, and 98% identical to those of strains MS1, MS2, MS3 and MK19. In summary, these data establish that strain JZL also belongs to the species *D. zeae*. To our knowledge, this is the first report indicating clivia as another natural host plant of *D. zeae*. Thereafter, the natural host range of *D. zeae* is expanded.

### *D. zeae* strains in Asian countries were usually isolated from monocots

*D. zeae* was reported to infect both monocots and dicots [7, 11]. In most cases, it was isolated from monocot natural hosts, such as maize, rice, banana, pineapple, *Brachiaria* and hyacinth [3, 10, 13, 14, 16, 18, 21, 23–26]. By analysing the distribution of *D. zeae*, we found that it was most geographically originated from southeast Asian countries, especially the southeast of China, on rice, maize and banana (Additional files 1 and 2). Apart from China, *D. zeae* was also isolated from Japan, South Korea, North Korea, Philippines, India, Indonesia and Bangladesh on rice plants, and from South Korea, Japan, Thailand and India on maize host. It was currently only found in China on banana host, and in Malaysia on pineapple (Additional file 2). Given that pineapple heart rot disease was also found in Philippines and Hawaii, the pathogen was probably a novel species different from *D. zeae* [21, 27]. These geographical distribution and natural host range findings suggest that *D. zeae* may have a high level of host specificity and geographic related evolution history.

### *D. zeae* strain JZL showed a significantly narrower host range than other *D. zeae* strains

To evaluate the host ranges of the *D. zeae* isolates, we performed pathogenicity tests on various reported hosts of *D. zeae*. Results showed that strains MS2 and MS3 could infect all the tested host plants similar to strain EC1, while JZL strains could not infect dicotyledonous plants including *Cucumis sativus*, *Benincasa hispida*, *Brassica pekinensis*, *Raphanus sativus*, *Daucus carota*, *Solanum tuberosm*, *Lycopersicon esculentum*, *Solanum melongena* and *Capsicum annuum*, but infect monocots including *Oryza sativa*, *Musa sapientum*, *Clivia miniata*, *Zingiber officinale*, *Gladiolus gandavensis*, *Colocasia esculenta* and *Alocasia macrorrhiza* (Table 1, Additional file 3), suggesting a significantly narrower host range than the other strains isolated from rice (strain EC1) and banana (strains MS2 and MS3). In addition, the soft rot symptoms on monocots caused by strains JZLs developed more slowly than those caused by the others except on clivia, and strains JZLs could not infect onion (*Allium cepa*) (Table 1, Additional file 3).

**Table 1:** Pathogenicity tests of *D. zeae* strains on some dicotyledonous and monocotyledonous plants

Inoculated plant			Inoculation amount, time	Diseased area (mm^2^)					
Class	Species	Organ		EC1	MS2	MS3	JZL1	JZL2	JLZ7
Dicots	*Cucumis sativus*	Fruit	2 μL, 24 h	57.22 ± 7.70	107.97 ± 42.00	65.46 ± 7.88	0	0	0
	*Benincasa hispida*	Fruit	2 μL, 24 h	1140.57 ± 15.55	1336.50 ± 3.32	1252.61 ± 18.71	0	0	0
	*Brassica pekinensis*	Petiole	2 μL, 12 h	56.81 ± 3.14	49.83 ± 2.73	42.40 ± 3.17	0	0	0
	*Raphanus sativus*	Tuber	2 μL, 24 h	156.89 ± 2.60	106.72 ± 2.03	99.84 ± 9.76	0	0	0
	*Daucus carota*	Tuber	2 μL, 24 h	274.83 ± 18.74	266.70 ± 8.98	231.42 ± 14.11	0	0	0
	*Solanum tuberosm*	Tuber	2 μL, 24 h	173.33 ± 4.28	211.05 ± 20.54	171.45 ± 6.07	0	0	0
	*Lycopersicon esculentum*	Fruit	100 μL, 2 d	908.22 ± 5.95	904.75 ± 9.80	945.81 ± 13.77	0	0	0
	*Solanum melongena*	Fruit	100 μL, 2 d	66.52 ± 2.95	61.83 ± 1.67	86.72 ± 0.85	0	0	0
	*Capsicum annuum*	Fruit	2 μL, 24 h	101.79 ± 8.81	106.65 ± 10.76	191.42 ± 10.55	0	0	0
Monocots	*Oryza sativa*	Stem	200 μL, 7 d	720.62 ± 21.48	575.71 ± 29.53	539.35 ± 17.77	452.67 ± 19.53	413.01 ± 12.64	499.85 ± 12.09
	*Musa sapientum*	Stem	200 μL, 7 d	1110.83 ± 19.23	1358.89 ± 17.78	1201.88 ± 19.91	407.90 ± 12.48	377.89 ± 13.71	464.23 ± 11.06
	*Clivia miniata*	Leaf	200 μL, 24 h	1643.86 ± 6.94	2195.85 ± 7.06	1239.95 ± 7.55	1807.41 ± 9.52	1693.21 ± 20.27	1594.90 ± 14.66
	*Allium cepa*	Bulb	2 μL, 24 h	185.83 ± 21.68	194.27 ± 18.73	119.90 ± 11.29	0	0	0
	*Zingiber officinale*	Tuber	2 μL, 24 h	134.37 ± 14.17	86.92 ± 6.75	87.31 ± 12.23	47.44 ± 7.16	65.81 ± 6.11	81.33 ± 13.96
	*Gladiolus gandavensis*	Stem	200 μL, 7 d	254.16 ± 9.05	168.17 ± 9.23	171.59 ± 5.92	36.75 ± 1.62	36.38 ± 2.39	36.85 ± 3.05
	*Colocasia esculenta*	Tuber	2 μL, 5 d	461.847 ± 15.55	247.31 ± 15.31	117.34 ± 8.00	81.83 ± 6.00	40.46 ± 5.54	79.48 ± 3.91
	*Alocasia macrorrhiza*	Stem	200 μL, 7 d	2860.90 ± 65.15	2763.17 ± 30.46	2633.75 ± 35.94	1316.86 ± 17.97	1430.45 ± 32.58	1381.58 ± 15.23

### *D. zeae* clivia strains showed weak aggressiveness on potato and cabbage, but were comparable with rice and banana strains on banana and clivia

**Fig. 3:**
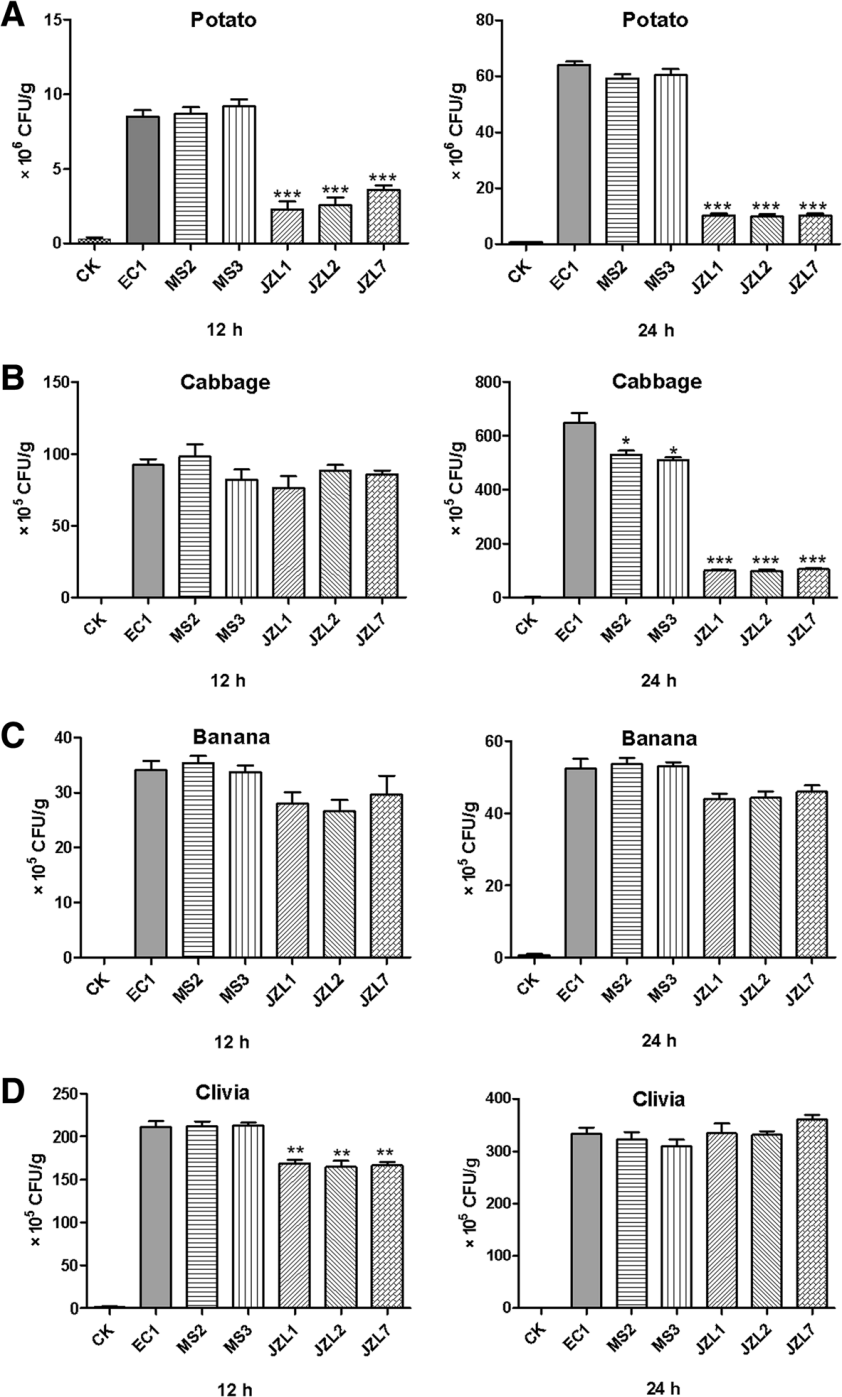
The number of bacterial cells of all the tested *D. zeae* strains invading into potato (**a**), cabbage (**b**), banana (**c**) and clivia (**d**), respectively. Healthy plant materials were surface-sterilized and inoculated with 2 μL of bacterial overnight cultures (OD_600_ = 2.0) in LB medium, and incubated at 28 °C. Tissues were taken out after 12 h and 24 h, respectively. And the diseased and surrounding healthy tissues in equal weight were cut and ground, and then added with 10 mL of sterilized 0.85% NaCl solution, stirred evenly, and 1 mL of which was diluted in series gradients, and 100 μL in each gradient was spread evenly onto LB agar plates in triplicates and kept at 28 °C for 24 h. Colonies between 30 to 300 CFU were counted. LB medium was used as a negative control. Each assay was repeated three times with duplicates

To understand whether the decreased virulence of *D. zeae* clivia strains on hosts was due to their weak aggressiveness, we performed pathogenicity tests on two dicotyledonous and two monocotyledonous plants in equal weight, and calculated the numbers of bacterial cells invading into the plant tissues. Results showed that the JZL strains could not propagate in potato or cabbage tissues after inoculation for 12 h and 24 h, while EC1, MS2 and MS3 strains grew rapidly in potato in 12 h, and slowly in cabbage (Fig. 3a and b). In banana tissues, the cell number of all the strains were similar no matter at 12 h or 24 h post inoculation (Fig. 3c). In clivia, JZL strains initially grew slower than strains EC1, MS2 and MS3, but caught up at 24 h post inoculation (Fig. 3d).

### *D. zeae* strains from clivia and banana differ in production of extracellular enzymes and polysaccharides

**Fig. 4:**
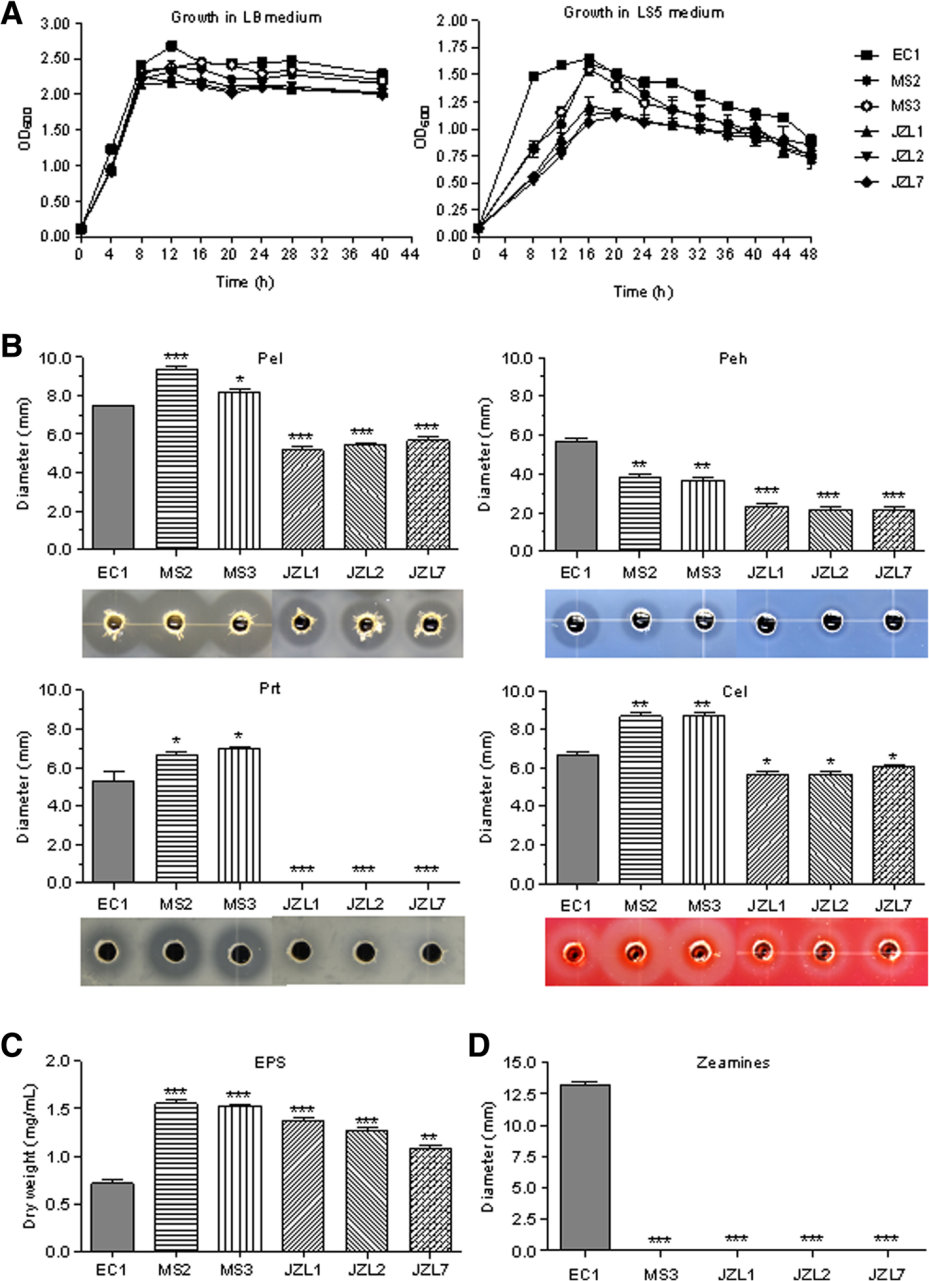
Major virulence factors produced by *D. zeae* strains. **a** Growth curves of *D. zeae* strains in LB and LS5 media. **b** Extracellular cell wall degrading enzymes produced by *D. zeae* strains. Samples of 40 μL bacterial cells (OD_600_ = 1.8) were added to the assay plate wells (4 mm in diameter) and incubated at 28 °C. Pel and Peh assay plates were treated with 4 N HCl after 11 h and 14 h respectively. Cel assay plate was stained with 0.1% (*w*/*v*) Congo Red for 15 min after 14 h, and decolored with 1 M NaCl twice. Prt assay plate was taken photos after 24 h without any further treatment. **c** Production of extracellular polysaccharides of *D. zeae* strains. Samples of 3 mL bacterial cultures (OD_600_ = 1.8) were applied into 300 mL LB medium and grown with shaking at 200 r/m for 12 h, which were centrifuged at 8000 rpm for 40 m, and then at 4000 rpm for 20 m to obtain 250 mL supernatants. Double volumes of absolute ethanol were added to the supernatants, mixed thoroughly, stored at 4 °C overnight for precipitation, and centrifuged at 8000 rpm for 40 m. Finally, supernatants were discarded and pellets were weighed after drying at 55 °C overnight. **d** Phytotoxins produced by *D. zeae* strains. The bioassay plate was prepared as previously described [44]. Samples of 20 μL of bacterial cultures (OD_600_ = 1.5 in LS5 medium) were added into the toxin bioassay plate wells (4 mm in diameter), and incubated overnight at 37 °C

From the above results of pathogenicity tests, we inferred that the narrower host range of JZL strains may be due to its less virulence than the other strains, and strains EC1, MS2 and MS3 probably possess some additional virulence factor(s) than the three JZL strains. To compare the major virulence factors produced by different *D. zeae* strains, we first measured their growth dynamic process. Results showed that all the strains shared a similar growth pattern reaching the peak from 12 h to 16 h both in LB and LS5 media, though the cell density of JZL strains could not reach OD_600_ = 1.3 in LS5 medium (Fig. 4a). We then measured the production of cell wall degrading enzymes and extracellular polysaccharides (EPS), which are two categories of virulence factors produced by the rice pathogen *D. zeae* strain EC1 [44, 59]. Results showed that JZL strains produced lower amount of pectate lyases (Pel), polygalacturonases (Peh), proteases (Prt) and cellulases (Cel) than the others, especially hardly any protein degrading zone was visible. In contrast, the two banana strains, MS2 and MS3, produced higher amounts of the above-mentioned extracellular enzymes than those produced by the rice strain EC1 except polygalacturonases (Fig. 4b). In a somewhat different pattern to the production of cell wall degradation enzymes, the rice pathogen strain EC1 produced significantly less EPS than the MS strains from banana and the JZL strains from clivia plants (Fig. 4c).

### *D. zeae* banana strain MS2 produces novel antibiotic-like toxin(s) different from zeamines with slightly weaker ability to inhibit rice seed germination and kill nematodes

Our previous study found that *D. zeae* rice strain EC1 produces zeamines as major virulence factors in both rice plants and potato tubers [11, 60]. The phytotoxins are encoded by a *zms* gene cluster only found in *D. zeae* rice strains including ZJU1202 [19] and DZ2Q [20], some *D. solani* and *Serratia plymuthica* strains [43, 61], but were absent in other *D. zeae* strains isolated from other hosts including strain MS1 from banana [12, 43]. The results from this study showed that JZL strains could not produce zeamines, neither could strain MS3 (Fig. 4d), consistent with our previous findings [43].

**Fig. 5:**
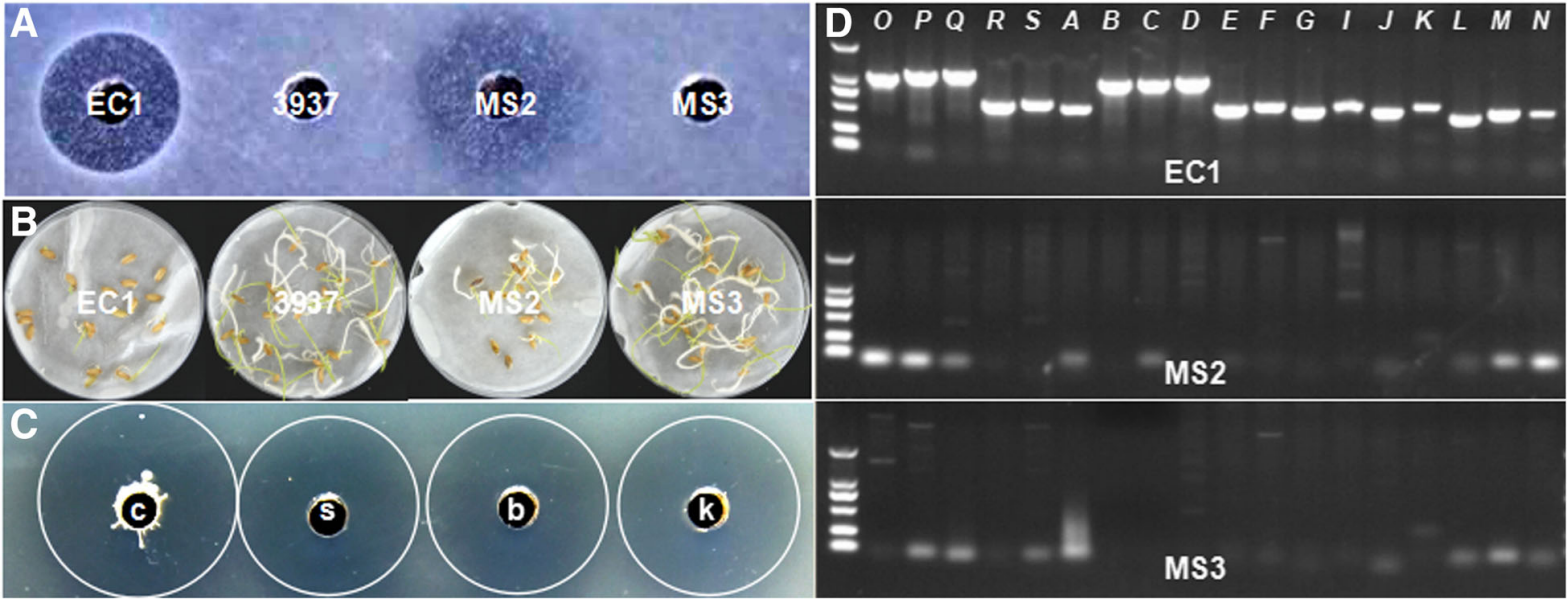
Phytotoxins produced by *Dickeya* strains. **a**, Bioassay of toxin production. **b**, Inhibitory activity of toxins from *Dickeya* strains against rice seed germination. Bacteria were grown in LB medium till OD_600_ = 1.5, and 20 seeds of rice variety CO39 were added to 5 ml of every bacterial culture and incubated at room temperature for 5 h, which were then cleaned and transferred onto a Petri dish with filter paper on it, and then incubated at 28 °C under 16 h light and 8 h dark conditions. Rice seeds incubated with same amount of *D. dadantii* 3937 were used as a control. **c**, The toxin produced by MS2 is an extracellular nonprotein metabolite. Strain MS2 was grown in LS5 medium till OD_600_ = 1.5 (c), and supernatant of the culture (s) was collected, which was treated by boiling at 100 °C for 10 min (b) or digestion with protease K at 37 °C for 30 min (k). Finally, the inhibition activity against the growth of *E. coli* DH5α was measured. **d**, PCR detection of zeamines biosynthesis genes from *zmsO* to *zmsN*. Based on the coding sequences of *zms* gene cluster in strain EC1 [43], 18 pairs of primers corresponding to *zmsO* to *zmsN* were designed to detect the zeamine biosynthsis gene cluster in *D. zeae* strains, which are presented in Additional file 4. The DNA marker is DL2000

Surprisingly, strain MS2 produced an antibiotic-like toxin(s) to inhibit the growth of *E. coli* DH5α, resulting in a halo with a vague edge, quite different from the halo with a clear edge caused by strain EC1 (Fig. 5a). Given that zeamines are able to inhibit rice seed germination, we tested the inhibitory activity of strain MS2 against rice seeds. The results indicated that strain MS2 had a moderate inhibitory effect on seed germination, with an inhibitory rate being about 50%, less than that of 87% by strain EC1 (Fig. 5b). Consistent with its negative antimicrobial activity phenotype (Fig. 3d, Fig. 5a), strain MS3 had no effect on seed germination (Fig. 5b). It is inferred that the inhibitory activity against seed germination is probably due to the toxin(s) produced by strain MS2, which is supposed to be a kind of extracellular nonprotein metabolite since it did not loss the activity after boiling at 100 °C for 10 min or treating with Protease K at 37 °C for 30 min (Fig. 5c).

To define whether the phytotoxin(s) produced by strain MS2 are zeamines or derivatives, we detected the zeamine biosynthesis genes from *zmsO* to *zmsN* based on their DNA sequences in strain EC1 [43], and results showed that no corresponding bands could be amplified if using the genomic DNA of strain MS2 or MS3 as a template (Fig. 5d). In combination with the sequencing results of MS2 genome (unpublished results), we confirmed that the phytotoxin(s) produced by *D. zeae* MS2 is probably novel, encoded by biosynthesis genes different from the *zms* gene cluster [43].

**Fig. 6:**
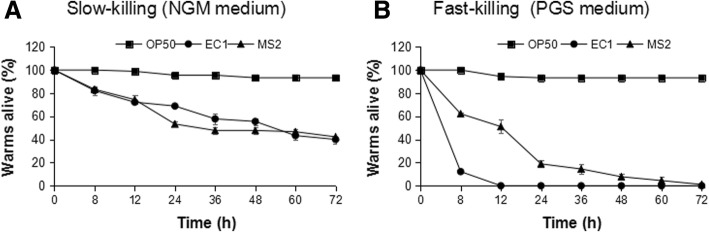
Worm-killing assay of *D. zeae* strains towards *C. elegans*. **a** Slow-killing; **b** Fast-killing. Strains EC1 and MS2 were grown in LB medium at 28 °C and the *C. elegans* food-source *E. coli* OP50 (negative control) at 37 °C overnight, and 50 μL of the liquid culture was spotted onto the center of NGM (slow-killing) (**a**) or PGS (fast-killing) (**b**) agar plates and allowed to dry thoroughly. In the slow-killing assay, 50 μM of floxuridine (FudR, Sigma) was added into NG agar to inhibit hatching of nematode eggs [58]. The plates containing bacteria were incubated at 28 °C and 37 °C respectively overnight and cooled for at least 2 h at room temperature before adding 30 L4 stage or adult hermaphrodite worms. The plates were kept at 20 °C, and live worms were scored

To investigate whether this phytotoxin(s) is toxic to nematodes, like zeamines produced by *S. plymuthica* A153 [62], we tested the nematode-killing dynamics of the two strains producing toxins. The results showed that both strains EC1 and MS2 had nematocidal activities, with similar slow-killing activity on NGM medium (Fig. 6a), whereas, strain EC1 had faster speed in killing *C. elegans* on PGS medium than strain MS2 (Fig. 6b). We inferred that the toxins produced by strains MS2 and EC1 may have similar killing effect on nematodes in the NGM medium. In the fast-killing assay, the worms treated with strain EC1 quickly became immobilized, and were completely dead in 12 h after treatment, while worms on strain MS2 lawn seemed more energetic and remained alive for a longer time compared with those on strain EC1 lawn, with only about 51% of worms died at 12 h, and 100% death occurred at 72 h after treatment (Fig. 6b), suggesting that the toxin(s) produced by strain MS2 was less toxic to *C. elegans* than zeamines produced by strain EC1 in the PGS medium.

Furthermore, the inhibition activity of *Dickeya* strains against some important pathogenic microorganisms was measured. Results showed that *D. dadantii* 3937 had little antimicrobial activity against all the tested microorganisms (Table 2). For the tested pathogenic bacteria, *D. zeae* EC1 showed the strongest antimicrobial effect with obvious antimicrobial halo zone on the pathogens except *Ralstonia solanacearum* EP1, while strains isolated from banana had little antibacterial activity except that strain MS2 inhibited the growth of *E. coli* DH5α (Table 2). The antifungal activity of EC1 was strongest, followed by strains MS2 and MS3, and the antagonistic activity of the *D. zeae* strains isolated from banana indicated that it is not the new toxin produced by MS2 that has the antifungal activity, since strain MS3 showed a similar antifungal activity as strain MS2 (Table 2).

**Table 2:** Inhibition activity of *Dickeya* strains against some pathogenic microorganisms

Microorganism	Description	Source or reference	Inhibition activity (mm)			
			EC1	MS2	MS3	3937
*Escherichia coli* DH5α	Indicator for toxin antagonism	Lab storage	5.23 ± 0.23	7.51 ± 0.34	0	0
*Ralstonia solanacearum* EP1	Pathogen of eggplant bacterial wilt	[68]	0	0	0	0
*Xanthomonas campestris* pv. *campestris* Xc1	Pathogen of crucifers black rot	[69]	9.68 ± 0.36	0	0	0
*Pseudomonas aeruginosa* PAO1	Pathogen of cystic fibrosis	[70]	4.04 ± 0.43	0	0	0
*Fusarium oxysporum* f.sp. *cubense* FOC4	Pathogen of banana wilt	[71]	6.17 ± 0.33	3.95 ± 0.39	3.32 ± 0.22	0
*Rhizoctonia solani* AG-1 IA	Pathogen of rice sheath blight	[72]	4.07 ± 0.23	1.31 ± 0.09	0.70 ± 0.15	0
*Magnaporthe oryzae* B157	Pathogen of rice blast	[73]	4.70 ± 0.28	1.98 ± 0.34	1.50 ± 0.31	0
*Peronophythora litchi*	Pathogen of litchi downy blight	[74]	11.69 ± 0.34	8.71 ± 0.15	3.48 ± 0.21	3.72 ± 0.22
*Colletotrichum capsici*	Pathogen of capsicum anthracnose	Lab storage	5.45 ± 0.14	5.26 ± 0.13	4.79 ± 0.10	0
*C. gloeosporioides*	Pathogen of mango anthracnose	Lab storage	4.91 ± 0.10	4.06 ± 0.20	3.59 ± 0.42	0
*Sporisorium scitamineum*	Pathogen of sugarcane smut	[75]	2.60 ± 0.06	2.27 ± 0.54	1.51 ± 0.22	0

### *D. zeae* JZL strains have stronger cell motility than other strains

**Fig. 7:**
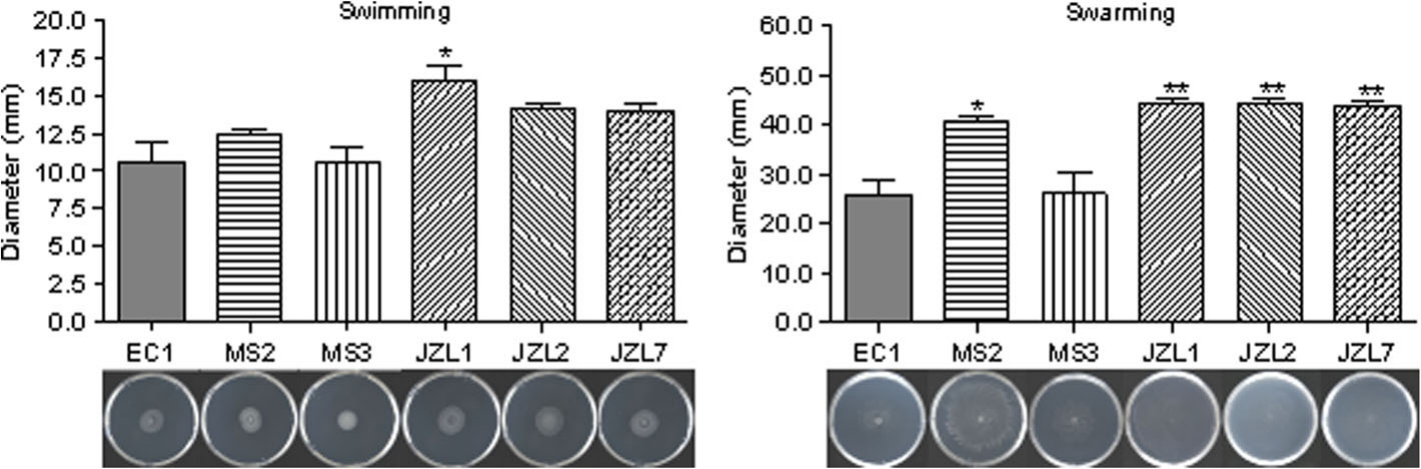
Cell motility of *D. zeae* strains. One microlitre of bacterial culture (OD_600_ = 1.5 in LB medium) was spotted onto the centre of a plate containing about 20 mL of semisolid swimming or swarming medium, which was then incubated at 28 °C for 20 h before measurement of the diameters of bacterial motility zone

Bacterial motility is of important pathological significance during the early stage of infection. In this study, the mobile capability of *D. zeae* strains was measured including swimming and swarming motility. Results showed that all the strains were capable of swimming and swarming, but in general, the strains isolated from clivia swarmed and swam faster than the other strains, except that strain JZL7 swarmed similar to strain MS2 (Fig. 7). Given that JZL strains were weaker than the other three strains in production of various virulence factors but showed comparable virulence against *Clivia miniata* (Fig. 4, Table 1, Additional file 3), we reasoned that the cell motility of JZL strains might contribute substantially in their pathogenicity against the clivia plants, which awaits further investigations.

## Discussion

*D. zeae* is a kind of pectinolytic bacteria causing soft rot disease on plants all over the world. It usually causes great agricultural economic losses in tropical, subtropical and temperate regions, especially in Southeast Asian countries such as Japan, Philippines, Bangladesh, China, India, Indonesia, South Korea, North Korea and Malaysia on maize, rice and banana (Additional file 1) [3, 14, 15, 19, 24, 26]. So far, the host range of *D. zeae* includes 25 dicotyledonous plants, and 18 monocotyledonous plants including the natural host clivia expanded in this study (Additional file 1).

By comparing the geographical distribution of *D. zeae*, we found that this species is centralized on monocot hosts in Asia (Additional file 1), similar to the situation that *D. solani* bacteria were mostly found in European countries and Israel on potato [10, 63]. The centralized distribution of *D. zeae* is probably due to the growth environment or host specificity related to bacterial evolution and adaptation during plant-pathogen interactions. According to our previous study, we found that the genome sequences of *D. zeae* rice strains EC1 and ZJU1202, both isolated from Guangdong Province, share 99.962% identity, while strain EC1 is only 95.863% identical to the rice strain DZ2Q isolated from Italy, and interestingly, all these three rice strains harbor an unique *zms* gene cluster encoding zeamines, which was absent in other sequenced *D. zeae* strains isolated from other sources [11, 43], suggesting that this *zms* gene cluster is probably related to their rice host specificity. In this study, we also found that strains MS2 and MS3, both isolated from banana, could not produce zeamines (Fig. 5), but strain MS2 produces another type of antimicrobial compounds with moderate inhibition activities on rice seed germination and nematicidal activity (Figs. 5 and 6), while strain MS3 produces none, in addition, strain MS2 showed higher pectinolytic activity, faster motility, and stronger virulence than strain MS3, indicating the complicated and dynamic process of *D. zeae* evolution.

We also isolated *D. zeae* strains from ornamental monocot plant *Clivia miniata* and validated them as the causal agents of clivia soft rot disease. Although MS2, MS3 and JZL strains are in a same big branch of *D. zeae*, they fall in different clades of the *16S rDNA* tree and the joint phylogenetic tree (Figs. 1 and 2). Given that *16S rDNA* gene sequence is insufficient to assign the taxonomy of a bacterium owing to its polyphyletic nature not only in a same family, but also in a same genus [64], we consider that the joint phylogenetic tree based on MLSA analysis is more reliable. Phenotypic characteristics of virulence factors indicated that *D. zeae* clivia strains JZLs produce remarkably less extracellular degrading enzymes than other strains, especially proteases (Fig. 4), probably responsible for the significantly narrower host range and the extremely less virulence on the tested hosts except clivia (Table 1). In contrast, *D. zeae* strains JZLs caused a comparable disease severity on clivia with the rice and banana strains (Table 1), which may suggest that these JZL strains have stronger affinity on their natural host plant than non-natural host.

The differences in production of virulence factors and bacterial aggressiveness among strains suggest that *D. zeae* strains causing soft rot disease are highly diverse whether from different sources or from the same host. Indeed, it has already been reported that *D. solani* strains from different climatic conditions, despite their genotypic homogeneity, behave very differently under particular environmental conditions, especially the incubation temperature [65]. The diversity in virulence factors and infection ability is not solely specific to certain species, rather, it is strain characteristic [66, 67].

The narrower host range and the lower pathogenicity of *D. zeae* JZL strains may be attributed to the loss of some important pathogenic islands during the evolution along with host clivia. Actually, in a long period of evolution and interaction with host plants, gene deletions and additional acquisition frequently occur, for instance, the *zms* gene cluster in *D. zeae* rice strains associated with the rice host specificity [11, 43, 60], and probably the genes encoding the antimicrobial compound(s) in strain MS2. Aggressiveness of *D. zeae* strains showed that EC1, MS2 and MS3 strains grew faster in potato and clivia than in cabbage and banana (Fig. 3), probably because the former two plants could provide more carbon sources for the bacterial growth. And the cell numbers of all the strains in banana tissues seemed comparable (Fig. 3c), while the disease degree caused by JZL strains was obviously lower than that caused by the other strains (Table 1, Additional file 3). We considered that the decreased virulence of JZL strains on monocotyledonous hosts is unrelated to the bacterial growth speed, which was also verified in Fig. 4a, but due to the reduced production of CWDEs (Fig. 4b).

Moreover, the antimicrobial compound(s) produced by strain MS2 is one of the major virulence factors inhibiting rice seed germination and killing nematodes. Genes encoding this new toxin(s) are being identified and the effects on rice seeds are being investigated. Whole genome comparison of these *Dickeya* zeae strains will be helpful for identification of the host-specific genes among *D. zeae* strains and revealing the interactions between pathogens and hosts.

## Conclusions

In this study, we identified the causal agents respectively from soft rot banana and clivia plants as *Dickeya zeae*. To our knowledge, this is the first time reporting clivia as the natural host of *D. zeae*. After comparing the virulence factors produced by different *D. zeae* strains isolated from rice, banana and clivia, we concluded that at least two types of *D. zeae* strains cause soft rot disease of banana, one of which produces a novel phytotoxin different from zeamines, while the other produces none, and the clivia isolates only infect monocots because of their weak aggressiveness on dicots. The virulence differentials of strains may provide targets for controlling bacterial soft rot diseases caused by *Dickeya*.

## Methods

### D. zeae strains collection, isolation and identification

Bacterial strains used in this study were listed in Additional file 4. *D. zeae* strain EC1 was isolated from diseased rice stem foot [11]. Strains MS2 and MS3 were isolated from the basal pseudostems of banana plants with soft rot disease respectively collected from Nansha and Panyu Districts in Guangzhou, Guangdong Province in 2012. *D. zeae* JZL strains were isolated from *Clivia miniata* (belonging to Amaryllidaceae) plants with soft rot symptoms at Fangcun flower market in Guangzhou city, Guangdong Province, China in 2017. *D. dadantii* 3937 isolated from African violets was also used for testing the virulence factors as a control.

MS2, MS3 and JZL strains that fulfilled Koch’s postulates were identified by sequencing of *16S rRNA* gene using 27F/1492R primers [46], and *atpD*, *gyrB*, *infB* and *rpoB* genes using primers and PCR conditions listed by Brady et al. [47]. DNA fragments were amplified using EasyTaq DNA polymerase (Transgen) and cloned into pUC19-T vector for sequencing. The sizes of the resultant amplicons were as follows: *16S rRNA*, 1507 bp; *atpD*, 884 bp; *gyrB*, 974 bp; *infB*, 1124 bp; *rpoB*, 1090 bp.

### Multilocus sequences analysis (MLSA) and phylogenetic analysis

Sequence similarities of all genes were determined by using BLASTn program, and all the sequences of related strains were obtained from GenBank database. Consensus sequences were aligned with ClustalW and trimmed in the following sizes: *16S rRNA*, 654 bp; *atpD*, 642 bp; *gyrB*, 745 bp; *infB*, 1042 bp; *rpoB*, 1000 bp. All the sequences of a same strain except *16S rRNA* were assembled for constructing a joint phylogenetic tree. Neighbor-joining trees were constructed by bootstrap analysis with 1000 replicates using MEGA5 package [48]. The GenBank accession numbers of *16S rRNA*, *atpD, gyrB*, *infB* and *rpoB* gene sequences of strain MS2 are MF973080, MG018807, MG018810, MG018813 and MG018816, respectively, those of strain MS3 are MF973081, MG018808, MG018811, MG018814 and MG018817, respectively, and those of strain JZL are MF973082, MG018809, MG018812, MG018815 and MG018818, respectively.

### Pathogenicity assay against monocotyledonous and dicotyledonous plants

The tested strains were grown in LB medium till OD_600_ = 1.5, and different plant organs of monocotyledons and dicotyledons listed in Table 1 were selected using different inoculation methods. For rice (*Oryza sativa*), banana (*Musa sapientum* ABB), *Gladiolus gandavensis* and *Alocasia macrorrhiza*, every 200 μL of bacterial cultures was injected into the basal stems of the seedlings, and into the bases of clivia leaves. For radish (*Raphanus sativus*), carrot (*Daucus carota*), potato (*Solanum tuberosm*), *Zingiber officinale*, and *Colocasia esculenta*, tubers were washed with tap water and dried with a paper towel, subsequently, surface-sterilized with 70% ethanol and then sliced evenly about 5 mm in thickness. Each slice was placed in a tray with moistened filter paper. Other plant materials to be inoculated were surface-sterilized. Bacterial cells of 2 μL were applied to the inoculated parts after piercing them with pipette tips except eggplant (*Solanum melongena*) and tomoto (*Lycopersicon esculentum*) inoculated with 100 μL of bacterial cultures. All trays were kept at 28 °C till symptoms appeared. Same volume of LB medium was inoculated as a negative control. Each assay was repeated three times with triplicates. The area of lesions were measured using Image J software.

### Tests on aggressiveness of D. zeae strains

The tested strains were grown in LB medium till OD_600_ = 2.0. Healthy potato tubers, cabbage petiole, banana pseudostems and clivia leaves were selected, washed with tap water, surface-sterilized with 70% ethanol, and placed onto the moistened filter paper in plates in biosafety cabinet under UV sterilization for 15 min. The plant tissues were pierced and inoculated with 2 μL of bacterial cultures. Plates were incubated at 28 °C. Same volume of LB medium was inoculated as a negative control. Tissues were taken out after 12 h and 24 h of incubation, respectively, where the diseased and surrounding healthy tissues in equal weight were cut and ground, then added with 10 mL of sterilized 0.85% NaCl solution, stirred evenly, and 1 mL of which was taken and diluted into 10^3^, 10^4^, and 10^5^ folds, and then 100 μL in each dilution gradient was spread evenly onto LB agar plates in triplicates. Plates were kept at 28 °C for 24 h, and colonies between 30 to 300 CFU (Colony-Forming Unit) were counted. Each assay was repeated three times with duplicates.

### Measurement of bacterial growth curves

Bacterial strains to be tested were grown in LB medium overnight at 28 °C. All the bacterial cultures were adjusted to OD_600_ = 2.0 and diluted into fresh LB medium in 1:100 ratio. Dilutions were mixed thoroughly and aliquots of 500 μL were transferred into 2.0 mL tubes. Bacteria were grown with shaking at 200 r/min under 28 °C and cell density was measured at 0, 4, 8, 12, 16, 20, 24, 28 and 40 h respectively. The experiment was repeated three times in triplicate.

### Measurement of cell wall degrading enzymatic (CWDE) activities

The activities of cell wall degrading enzymes were measured using the medium recipe described previously [49–51]. Briefly, assay medium was prepared, and 30 mL of each medium was poured into a 12 × 12 cm square plate. Subsequently, wells (4 mm in diameter) were punched after solidification. Samples of 40 μL bacterial cells were added into the wells after they had grown to OD_600_ = 1.8. All plates were incubated at 28 °C. Pectate lyase (Pel) and polygalacturonase (Peh) assay plates were covered with 4 N HCl after 11 h and 14 h respectively. Cellulase (Cel) assay plate was stained with 0.1% (*w*/*v*) Congo Red for 15 min after 14 h, and treated with 1 M NaCl twice. Protease (Prt) activity was indicated by the transparent halos surrounding the wells after 24 h incubation. The experiment was repeated three times with duplicates.

### Assay of extracellular polysaccharide (EPS) production

Extracellular polysaccharides (EPS) are one of the important virulence factors for bacterial phytopathogens and the main toxic factors leading to water-logging and wilting on plants after infection [52–54]. For measuring the production of EPS, single colonies of the tested strains were picked and transferred into 10 mL LB medium for culture overnight at 28 °C until OD_600_ = 1.8, afterwards, 3 mL of which was applied into 300 mL LB medium, and grown with shaking at 200 r/m for 12 h. Cultures were centrifuged at 8000 rpm for 40 m, and then at 4000 rpm for 20 m to obtain 250 mL supernatants. Double volumes of absolute ethanol were added to the supernatants, mixed thoroughly, stored at 4 °C overnight for precipitation, and subjected to centrifugation at 8000 rpm for 40 m. Finally, supernatants were discarded and pellets were weighed after drying at 55 °C overnight. The experiment was repeated three times in triplicate.

### Toxin bioassay

The tested strains were streaked on LB plates and single colonies were transferred into LS5 medium [55] to grow at 28 °C overnight. Cell density was adjusted to OD_600_ = 1.5 prior to bioassay. The bioassay plate was prepared as previous described [44]. Wells of 4 mm in diameter were punched in the plate and 20 μL of the bacterial cultures were added into the wells. The plate was incubated overnight at 37 °C.

To preliminarily determine the chemical property of the toxin, supernatant of strain MS2 overnight culture was collected, and treated by boiling at 100 °C for 10 min and by digestion with protease K at 37 °C for 30 min, respectively. Finally, the inhibition activity against the growth of *E. coli* DH5α was measured. The experiment was repeated three times in triplicate.

### Inhibition of phytotoxins against rice seed germination

Bacteria were grown in LB medium till OD_600_ = 1.5, and 20 seeds of rice variety CO39 were added to 5 ml of every bacterial culture and kept at room temperature for 5 h, then taken out to clean in sterilized water and transferred onto the moistened filter paper in a Petri dish, which was then kept at 28 °C under 16 h light and 8 h dark conditions. Rice seeds incubated with same amount of *D. dadantii* 3937 were used as a control. The experiment was repeated three times in triplicate.

### Detection of zeamine biosynthesis gene cluster

Based on the coding sequences of *zms* gene cluster in strain EC1 [43], we designed 18 pairs of primers (Additional file 5) corresponding to *zmsO* to *zmsN* to detect the zeamine biosynthsis gene cluster in *D. zeae* strains, which are presented in Additional file 4. DNA fragments were amplified using EasyTaq DNA polymerase (Transgen) using conditions as following: 95 °C, 2 min; 30 cycles of 94 °C, 30 s, 55 °C, 30 s and 72 °C, 1 min; 72 °C, 5 min.

### Antimicrobial activity assay

Pathogenic microorganisms used in this study were listed in Table 2, in which, bacterial pathogens were grown in LB medium overnight and fungi were grown on PDA medium at 28 °C until colonies covered the Petri dishes. For antibacterial activity, the same method of toxin bioassay against *E. coli* described in previous section was used against other bacterial pathogens, and the diameters of the antibacterial halos were measured. For antifungal activity, fungal dishes in diameter of 4 mm were punched and placed onto the center of PDA plate, and 2 μL of EC1, MS2, MS3 and 3937 strain overnight cultures were respectively spotted onto each of the four sides of the fungal dishes (3 cm away from the edge of the fungal dishes). Plates were incubated at 28 °C until fungal colonies on blank plates covered the petri dishes, and the distance between bacterial colonies and the hyphal edge of the tested fungi was measured. The experiment was repeated three times.

### Nematode killing activity

Wild type *Caenorhabditis elegans* were maintained according to the methods as previously described [56], and the medium recipe for worm-killing assays was referred to in literature reported by Tan et al. [57]. *D. zeae* strains EC1 and MS2 were routinely grown in LB medium at 28 °C and the *C. elegans* food-source *E. coli* OP50 at 37 °C overnight, and 50 μL of the liquid culture was spotted onto the center of PGS (fast-killing) or NGM (slow-killing) agar plates and allowed to dry thoroughly. In the slow-killing assay, 50 μM of floxuridine (FudR, Sigma) was added into NG agar to inhibit hatching of nematode eggs [58]. *E. coil* OP50 culture was used as a negative control. The plates containing bacteria were incubated at 28 °C and 37 °C respectively overnight and cooled for at least 2 h at room temperature before adding 30 L4 stage or adult hermaphrodite worms. The plates were kept at 20 °C, and live worms were scored. Each trial was repeated three times in triplicate.

### Measurement of cell motility

To determine the cell motility, media for swimming (per litre contains 10 g bactotryptone, 5 g NaCl and 3 g agar) and swarming (per litre contains 5 g peptone, 3 g yeast extract and 4 g agar) assay were prepared. One microlitre of overnight bacterial culture (OD_600_ = 1.5) was spotted onto the centre of a plate containing about 20 mL of .each medium. The plates were incubated at 28 °C for 20 h before measurement of the diameters of bacterial motility zone. Each experiment was repeated at least three times in triplicate [44]*.*

### Statistic analysis

All the experiments were repeated in three times with duplicates or triplicates. For statistic analysis, GraphPad Prism 5.0 software was used to performed Student’s *t*-test, and the data of *D. zeae* strains were normalized to those of strain EC1. * indicates *P* < 0.05, ** indicates *P* < 0.001, and *** indicates *P* < 0.0001.

## Additional files

Additional file 1: The hosts and origins of *D. zeae* strains. (DOC 92 kb)

Additional file 2: Natural hosts and distribution of *D. zeae* strains in southeast Asia. The map was drawn using Photoshop CS6 software and host plant icons were added on the corresponding locations of the map. (TIF 2891 kb)

Additional file 3: The diseased symptoms of the tested strains on dicotyledonous and monocotyledonous hosts corresponding to Table 1. (PDF 6199 kb)

Additional file 4: Bacterial strains used in this study. (DOC 36 kb)

Additional file 5: Primers used in this study for detection of zeamines biosynthesis genes. (DOC 54 kb)

## Abbreviations

AHL: Acyl homoserine lactone
Cel: Cellulases
CWDE: Cell wall degrading enzyme
EPS: Extracellular polysaccharides
MLSA: Multilocus sequences analysis
Peh: Polygalacturonases
Pel: Pectate lyases
Prt: Proteases
QS: Quorum sensing

## References

[1] Liu G (2007). List of Plant Quarantine Pests in the People’s Republic of China. Pestic Mark Inf.

[2] Mansfield J, Genin S, Magori S, Citovsky V, Sriariyanum M, Ronald P, Dow M, Verdier V, Beer SV, Machado MA, Toth I, Salmond G, Foster GD (2012). Top 10 plant pathogenic bacteria in molecular plant pathology. Mol Plant Pathol.

[3] Samson R, Legendre JB, Christen R, Fischer-Le Saux M, Achouak W, Gardan L (2005). Transfer of *Pectobacterium chrysanthemi* (Burkholder et al. 1953) Brenner et al. 1973 and *Brenneria paradisiaca* to the genus *Dickeya* gen. nov. as *Dickeya chrysanthemi* comb. nov. and *Dickeya paradisiaca* comb. nov. and delineation of four novel species, *Dickeya dadantii* sp. nov., *Dickeya dianthicola* sp. nov., *Dickeya dieffenbachiae* sp. nov. and *Dickeya zeae* sp. nov. Int J Syst Evol Microbiol.

[4] Brady C, Cleenwerck I, Denman S, Venter SN, Rodriguez-Palenzuela P, Coutinho TA, Devos P (2012). Proposal to reclassify *Brenneria quercina* (Hildebrand and Schroth 1967) Hauben et al. 1999 into a new genus, *Lonsdalea gen*. nov., as *Lonsdalea quercina* comb. nov., descriptions of *Lonsdalea quercina* subsp. quercina comb. nov., *Lonsdalea quercina* subsp. iberica subsp. nov. and *Lonsdalea quercina* subsp. britannica subsp. nov., emendation of the description of the genus Brenneria, reclassification of *Dickeya dieffenbachiae* as *Dickeya dadantii* subsp. *dieffenbachiae* comb. nov., and emendation of the description of *Dickeya dadantii*. Int J Syst Evol Microbiol.

[5] Parkinson N, Devos P, Pirhonen M, Elphinstone J (2014). *Dickeya aquatica* sp nov., isolated from waterways. Int J Syst Evol Microbiol.

[6] Tian Y, Zhao Y, Yuan X, Yi J, Fan J, Xu Z, Hu B, De Boer SH, Li X (2016). *Dickeya fangzhongdai* sp. nov., a plant-pathogenic bacterium isolated from pear trees (*Pyrus pyrifolia*). Int J Syst Evol Microbiol.

[7] Hussain MB, Zhang HB, Xu JL, Liu Q, Jiang Z, Zhang LH (2008). The acyl-homoserine lactone-type quorum-sensing system modulates cell motility and virulence of *Erwinia chrysanthemi* pv. *zeae*. J Bacteriol.

[8] Sławiak M, van Beckhoven JRCM, Speksnijder AGCL, Czajkowski R, Grabe G, van der Wolf JM (2009). Biochemical and genetical analysis reveal a new clade of biovar 3 *Dickeya* spp. strains isolated from potato in Europe. Eur J Plant Pathol.

[9] Lin BR, Shen HF, Pu XM, Tian XS, Zhao WJ, Zhu SF, Dong MM (2010). First report of a soft rot of banana in Mainland China caused by a *Dickeya* sp. (*Pectobacterium chrysanthemi*). Plant Dis.

[10] Toth IK, van der Wolf JM, Saddler G, Lojkowska E, Hélias V, Pirhonen M, Tsror L, Elphinstone JG (2011). *Dickeya* species: an emerging problem for potato production in Europe. Plant Pathol.

[11] Zhou JN, Zhang HB, Wu J, Liu QG, Xi PG, Lee J, Liao JL, Jiang ZD, Zhang LH (2011). A novel multi-domain polyketide synthase is essential for zeamine antibiotics production and the virulence of *Dickeya zeae*. Mol Plant-Microbe Interact.

[12] Zhang JX, Shen HF, Pu XM, Lin BR (2014). Identification of *Dickeya zeae* as a casual agent of bacterial soft rot in banana in China. Plant Dis.

[13] Sinha SK, Prasad M (1977). Bacterial stalk rot of maize, its symptoms and host-range. Zentralbl Bakteriol Parasitenkd Infektionskr Hyg.

[14] Goto M (1979). Bacterial Foot Rot of Rice Caused by a Strain of *Erwinia-Chrysanthemi*. Phytopathology.

[15] Liu QG, Wang ZZ (2004). Infection characteristics of *Erwinia chrysanthemi* pv. *zeae* on rice. J S China Agric Univ.

[16] Jafra S, Przysowa J, Gwizdek-Wiśniewska A, van der Wolf JM (2008). Potential of bulb-associated bacteria for biocontrol of hyacinth soft rot caused by *Dickeya zeae*. J Appl Microbiol.

[17] Stead DE, Parkinson N, Bew J, Hennessy J, Wilson JK, Elphinstone JE (2010). The first record of *Dickeya zeae* in the UK. Plant Pathol.

[18] Myung IS, Jeong IH, Moon SY, Kim WG, Lee SW, Lee YH, Lee YK, Shim HS, Ra DS (2010). First report of bacterial stalk rot of sweet corn caused by *Dickeya zeae* in Korea. New Dis Rep.

[19] Li B, Shi Y, Ibrahim M, Liu H, Shan C, Wang Y, Kube M, Xie GL, Sun G (2012). Genome sequence of the rice pathogen *Dickeya zeae* strain ZJU1202. J Bacteriol.

[20] Bertani I, Passos da Silva D, Abbruscato P, Piffanelli P, Venturi V (2013). Draft genome sequence of the plant pathogen *Dickeya zeae* DZ2Q, isolated from rice in Italy. Genome Announc.

[21] Marrero G, Schneider KL, Jenkins DM, Alvarez AM (2013). Phylogeny and classification of *Dickeya* based on multilocus sequence analysis. Int J Syst Evol Microbiol.

[22] Pritchard L, Saddler GS, Parkinson NM, Bertrand V, Elphinstone JG (2012). Detection of phytopathogens of the genus *Dickeya* using a PCR primer prediction pipeline for draft bacterial genome sequences. Plant Pathol.

[23] Pritchard L, Humphris S, Saddler GS, Elphinstone JG, Pirhonen M, Toth IK (2013). Draft genome sequences of 17 isolates of the plant pathogenic bacterium dickeya. Genome Announc.

[24] Zhang JX, Lin BR, Shen HF, Pu XM (2013). Genome sequnence of the banana pathogen *Dickeya zeae* strain MS1, which causes bacteria soft rot. Genome Announc.

[25] Martinez-Cisneros BA, Juarez-Lopez G, Valencia-Torres N, Duran-Peralta E, Mezzalama M (2014). First report of bacterial stalk rot of maize caused by *Dickeya zeae* in Mexico. Plant Dis.

[26] Ramachandran K, Manaf UA, Zakaria L (2015). Molecular characterization and pathogenicity of *Erwinia* spp. associated with pineapple [*Ananas comosus* (L.) Merr.] and papaya (*Carica papaya* L.). J Plant Protection Res.

[27] Kumar A, Hunjan MS, Kaur H, Dhillon HK, Singh PP (2017). Biochemical responses associated with resistance to bacterial stalk rot caused by *Dickeya zeae* in maize. J Phytopathol.

[28] Reifschneider FJB, Lopes CA (1982). Bacterial top and stalk rot of maize *Zea mays* in brazi. Plant Dis.

[29] Masumi M, Izadpanah K (1988). Occurrence of bacterial stalk rot of maize in Fars Province. Iranian J Plant Pathol.

[30] Takeuchi T, Kodama F (1992). Bacterial stalk rot of corn caused by *Erwinia chrysanthemi* pv. *zeae* (Sabet) Victoria, Arboleda et Muñoz occurred in Hokkaido, Japan.

[31] Zheng YN (2006). Occurrence and control of bacterial stalk rot of maize. J Anhui Agric Sci.

[32] El-Helaly AF, Abo-El-Dahab MK, El-Goorani MA, Gabr MR (1978). Identification of *Erwinia* sp., causing stalk rot of maize in Egypt. Zentralbl Bakteriol Naturwiss.

[33] Wei G, Huang YL, Huang XS (1986). Infection way and hosts of rice foot rot bacteria. Guangxi Agric Sci.

[34] Liu QG, Zhang Q, Wei CD (2013). Advances in Research of Rice Bacterial Foot Rot. Sci Agric Sin.

[35] Zhou Y, Zhai YC, Cao BH (1989). Rice bacterial foot rot seriously happened in Rudong County, Jiangsu Province. Plant Prot.

[36] Yang MH (2000). Serious rice bacterial foot rot occurred in Taining County of Fujian Province in 1999. Plant Prot Technol Ext.

[37] Li CY (2007). Occurrence and control measures of rice bacterial foot rot in Anshun. Plant Doct.

[38] Xue NQ, Liu Y (2008). Occurrence and control of rice bacterial foot rot. Shandong Agric Sci.

[39] Collmer A, Bauer DW (1994). *Erwinia chrysanthemi* and *Pesudominas syringae*: plant pathogens trafficking in extracellular virulence proteins. Curr Top Microbiol Immunol.

[40] Reverchon S, Rouanet C, Expert D, Nasser W (2002). Charaterization of indigoidine biosynthetic genes in *Erwinia chrysanthemi*. Mol Microbiol.

[41] Franza T, Mahé B, Expert D (2005). *Erwinia chrysanthemi* requires a second iron transport route dependent of the siderophore achromobactin for extracellular growth and plant infection. Mol Microbiol.

[42] Yap MN, Yang CH, Barak JD, Jahn CE, Charkowski AO (2005). The *Erwinia chrysanthemi* type III secretion system is required for multicellular behavior. J Bacteriol.

[43] Zhou JN, Cheng YY, Lv MF, Liao LS, Chen YF, Gu YF, Liu SY, Jiang ZD, Xiong YY, Zhang LH (2015). The complete genome sequence of *Dickeya zeae* EC1 reveals substantial divergence from other *Dickeya* strains and species. BMC Genomics.

[44] Zhou JN, Zhang HB, Lv MF, Chen YF, Liao LS, Cheng YY, Liu SY, Chen SH, He F, Cui ZN, Jiang ZD, Chang CQ, Zhang LH (2016). SlyA regulates phytotoxin production and virulence in *Dickeya zeae* EC1. Mol Plant Pathol.

[45] Lv MF, Chen YF, Liao LS, Liang ZB, Shi ZR, Tang YX, Ye SX, Zhou JN, Zhang LH (2018). Fis is a global regulator critical for modulation of virulence factor production and pathogenicity of *Dickeya zeae*. Sci Rep.

[46] Coenye T, Falsent E, Vananneyt M, Hostef B, Govant JRW, Kersters K, Vandamme P (1999). Classification of *Alcaligenes faecalis*-like isolates from the environment and human clinical samples as *Ralstonia gilardii* sp. nov. Int. J Syst Bacteriol.

[47] Brady C, Cleenwerck I, Venter SN, Vancanneyt M, Swings J, Coutinho TA (2008). Phylogeny and identification of *Pantoea* species associated with plants, humans and the natural environment based on multilocus sequence analysis (MLSA). Syst Appl Microbiol.

[48] Tamura K, Peterson D, Peterson N, Steche G, Nei M, Kumar S (2011). MEGA5: molecular evolutionary genetics analysis using maximum likelihood, evolutionary distance, and maximum parsimony methods. Mol Biol Evol.

[49] Barras F, Thurn KK, Chatterjee AK (1987). Resolution of four pectate lyase structural genes of *Erwinia chrysanthemi* (EC16) and characterization of the enzymes produced in *Escherichia coli*. Mol Gen Genet.

[50] Scott-Craig JS, Panaccione DG, Cervone F, Walton JD (1990). Endopolygalacturonase is not required for pathogenicity of *Cochliobolus carbonum* on maize. Plant Cell.

[51] Chatterjee A, Cui Y, Liu Y, Dumenyo CK, Chatterjee AK (1995). Inactivation of *rsmA* leads to overproduction of extracellular pectinases, cellulases, and proteases in *Erwinia carotovora* subsp. *carotovora* in the absence of the starvation/cell density-sensing signal, *N*-(3-oxohexanoyl)-L-homoserine lactone. Appl Environ Microbiol.

[52] Hayward AC (1991). Biology and epidemiology of bacterial wilt caused by *Pseudomonas solanacearum*. Annu Rev Phytopathol.

[53] Kao CC, Barlow E, Sequeira L (1992). Extracellular polysaccharide is required of wild-type virulence of *Pseudomonas solanacearum*. J Bacteriol.

[54] Condemine G, Castillo A, Passeri F, Enard C (1992). The PecT repressor coregulates synthesis of exopolysaccharides and virulence factors in *Erwinia chrysanthemi*. Mol Plant-Microbe Interact.

[55] Liao LS, Cheng YY, Liu SY, Zhou JN, An SW, Lv MF, Chen YF, Gu YF, Chen SH, Zhang LH (2014). Production of novel antibiotics zeamines through optimizing *Dickeya zeae* fermentation conditions. PLoS One.

[56] Stiernagle T, Fay D (1999). Maintenance of *C. elegans*. *C. elegans*: a practical approach.

[57] Tan MW, Mahajan-Miklos S, Ausubel FM (1999). Killing of *Caenorhabditis elegans* by *Pseudomonas aeruginosa* used to model mammalian bacterial pathogenesis. Pro Natl Acad Sci USA.

[58] Houthoofd K, Braeckman BP, Vanfleteren JR (2004). The hunt for the record life span in *Caenorhabditis elegans*. J Gerontol A-Biol Sci Med Sci.

[59] Chen YF, Lv MF, Liao LS, Gu YF, Liang ZB, Shi ZR, Liu SY, Zhou JN, Zhang LH (2016). Genetic modulation of c-di-GMP turnover affects multiple virulence traits and bacterial virulence in rice pathogen *Dickeya zeae*. PLoS One.

[60] Cheng YY, Liu XL, An SW, Chang CQ, Zou YQ, Huang LH, Zhong J, Liu QG, Jiang ZD, Zhou JN, Zhang LH (2013). A nonribosomal peptide synthase containing a stand-Alone condensation domain is essential for phytotoxin zeamine biosynthesis. Mol Plant-Microbe Interact.

[61] Masschelein J, Mattheus W, Gao LJ, Moons P, Van Houdt R, Uytterhoeven B, Lamberigts C, Lescrinier E, Rozenski J, Herdewijn P, Aertsen A, Michiels C, Lavigne R (2013). A PKS/NRPS/FAS hybrid gene cluster from *Serratia plymuthica* RVH1 encoding the biosynthesis of three broad spectrum, zeamine-related antibiotics. PLoS One.

[62] Hellberg JEEU, Matilla MA, Salmond GPC (2015). The broad-spectrum antibiotic, zeamine, kills the nematode worm *Caenorhabditis elegans*. Front Microbiol.

[63] van der Wolf JM, Nijhuis EH, Kowalewska MJ, Saddler GS, Parkinson N, Elphinstone JG, Pritchard L, Toth IK, Lojkowska E, Potrykus M, Waleron M, de Vos P, Cleen-Werck I, Pirhonen M, Garlant L, Helias V, Pothier JF, Pflüger V, Duffy B, Tsror L, Manulis S (2014). *Dickeya solani* sp. nov., a pectinolytic plant pathogenic bacterium isolated from potato (*Solanum tuberosum*). Int J Syst Evol Microbiol.

[64] Liu SY, Tang YX, Wang DC, Lin NQ, Zhou JN (2016). Identification and characterization of a new Enterobacter onion bulb decay caused by *Lelliottia amnigena* in China. App Micro Open Access.

[65] Golanowska M, Kielar J, Łojkowska E (2017). The effect of temperature on the phenotypic features and the maceration ability of Dickeya solani strains isolated in Finland, Israel and Poland. Eur J Plant Pathol.

[66] Potrykus M, Golanowska M, Hugouvieux-Cotte-Pattat N, Lojkowska E (2014). Regulators involved in *Dickeya solani* virulence, genetic conservation, and functional variability. Mol Plant-Microbe Interact.

[67] Alič Š, Naglič T, Tušek-Žnidarič M, Peterka M, Ravnika M, Dreo T (2017). Putative new species of the genus *Dickeya* as major soft rot pathogens in *Phalaenopsis* orchid production. Plant Pathol.

[68] Li P, Wang DC, Yan JL, Zhou JN, Deng YY, Jiang ZD, Cao BH, He ZF, Zhang LH (2016). Genomic analysis of phylotype I strain EP1 of *Ralstonia solanacearum* species complex reveals substantial divergence from other *Ralstonia solanacearum* strains. Front Microbiol.

[69] Deng YY, Wu J, Yin WF, Li P, Zhou JN, Chen SH, He F, Cai J (2016). Diffusible signal factor family signals provide a fitness advantage to *Xanthomonas campestris* pv. *campestris* in interspecies competition. Environ Microbiol.

[70] Zhou L, Wang J, Zhang LH (2007). Modulation of bacterial Type III secretion system by a spermidine transporter dependent signaling pathway. PLoS One.

[71] Li MH, Xie XL, Lin XF, Shi JX, Ding Z, Ling JF, Xi PG, Zhou JN, Leng YQ, Zhong SB, Jiang ZD (2014). Functional characterization of the gene FoOCH1 encoding a putative 4 a-1, 6-mannosyltransferase in *Fusarium oxysporum* f. sp. *cubense*. Fungal Genet Biol.

[72] Shu CW, Zou CJ, Chen JL, Tang F, Yi RH, Zhou EX (2014). Genetic diversity and population structure of *Rhizoctonia solani* AG-1 IA, the causal agent of rice sheath blight, in South China. Can J Plant Pathol.

[73] Zhang SL, Liang ML, Naqvi NI, Lin CX, Qian WQ, Zhang LH, Deng YZ (2017). Phototrophy and starvation-based induction of autophagy upon removal of Gcn5-catalyzed acetylation of Atg7 in *Magnaporthe oryzae*. Autophagy.

[74] Liao LS, Zhou JN, Wang HS, He F, Liu SY, Jiang ZD, Chen SH, Zhang LH (2015). Control of litchi downy blight by zeamines produced by *Dickeya zeae*. Sci Rep.

[75] Liu SY, Lin NQ, Chen YM, Liang ZB, Liao LS, Lv MF, Chen YF, Tang YX, He F, Chen SH, Zhou JN, Zhang LH (2017). Biocontrol of sugarcane smut disease by interference of fungal sexual mating and hyphal growth using a bacterial isolate. Front Microbiol.

