# Supplementary material for: Dickeya zeae strains isolated from rice, banana and clivia rot plants show great virulence differentials
Source: BMC Microbiol. 2018 Oct 18;18:136. doi: 10.1186/s12866-018-1300-y (PMC6194671; doi:10.1186/s12866-018-1300-y)
Supplement: Supplementary file 1 — The hosts and origins of D. zeae strains. (DOC 92 kb) [file 12866_2018_1300_MOESM1_ESM.doc]

**Additional file 1. The hosts and origins of *D. zeae* strains.**

| **Host or biological origin** | | **Geographical origin** | **Way of infection** | **Reference** |
| --- | --- | --- | --- | --- |
| Dicots | Potato | Australia, Papua New Guinea | Natural infection | [3,10,23] |
|  | Tobacco | Cuba | Natural infection | [3] |
|  | *Chrysanthemum* | USA, UK | Natural infection | [3,17] |
|  | *Philodendron* | Greece, USA, Florida | Natural infection | [3,22] |
|  | Sugarbeet | India | Artificial inoculation | [13,27] |
|  | Tomato | India | Artificial inoculation | [13] |
|  | Sweet potato | India | Artificial inoculation | [13,27] |
|  | Cabbage | China | Artificial inoculation | [12,28] |
|  | Carrot | China | Artificial inoculation | [12,28] |
|  | Papaya | India | Artificial inoculation | [13,27] |
|  | Cucumber | China | Artificial inoculation | [12] |
|  | Towel gourd | China | Artificial inoculation | [12] |
|  | *Balsamine* | China | Artificial inoculation | [28] |
|  | Bean | India | Artificial inoculation | **[27]** |
|  | Turnip | India | Artificial inoculation | **[27]** |
|  | Radish | India | Artificial inoculation | **[27]** |
|  | Eggplant | India | Artificial inoculation | **[27]** |
|  | Chilli | India | Artificial inoculation | **[27]** |
|  | Peanut | China | Artificial inoculation | [28] |
|  | *Kalanchoe thyrsiflora* | China | Artificial inoculation | [29] |
|  | *Aeonium* | China | Artificial inoculation | [29] |
|  | *Gymnocalycium* | China | Artificial inoculation | [29] |
|  | African violet | China | Artificial inoculation | [29] |
|  | *Dianthus* | China | Artificial inoculation | [29] |
|  | Poinsettia | China | Artificial inoculation | [29] |
| Monocots | Maize | USA, Brazil, France, Italy, Senegal, Cuba, Egypt, Mexico, India, Korea, Iran, Japan, China, Thailand | Natural infection | [3,13,18,23,25,27,63-67] |
|  | Rice | Japan, Philippines, Bangladesh, China, India, Indonesia, South Korea, North Korea, Italy | Natural infection | [10,14,15,19,20] |
|  | Banana | Ivory Coast, Jamaica, Panama, Martinique, China | Natural infection | [3,24] |
|  | Pineapple | Martinique, Queensland, Malaysia | Natural infection | [[21,](http://xueshu.baidu.com/s?wd=author:(Kogeethavani Ramachandran) &tn=SE_baiduxueshu_c1gjeupa&ie=utf-8&sc_f_para=sc_hilight=person)26] |
|  | *Brachiaria* | Guyana | Natural infection | [3] |
|  | Hyacinth | Netherlands | Natural infection | [16] |
|  | Clivia | China | Natural infection | This study |
|  | Sugarcane | China | Artificial inoculation | [28] |
|  | Sorghum | India | Artificial inoculation | [13,27] |
|  | *Belamcanda* | China | Artificial inoculation | [28] |
|  | *Hemerocallis* | China | Artificial inoculation | [28] |
|  | *Pennisetum* | India | Artificial inoculation | **[27]** |
|  | *Dieffenbachia* | China | Artificial inoculation | [29] |
|  | *Syngonium* | China | Artificial inoculation | [29] |
|  | Monstera | China | Artificial inoculation | [29] |
|  | Iris | China | Artificial inoculation | [28] |
|  | Onion | India, China | Artificial inoculation | [13,27] |
|  | Taro | China | Artificial inoculation | [29] |
| / | Water | Israel, New South Wales, Scotland, England | Not appropriate | [3,23] |
