# Supplementary figures and images for: Dickeya zeae strains isolated from rice, banana and clivia rot plants show great virulence differentials
Source: BMC Microbiol. 2018 Oct 18;18:136. doi: 10.1186/s12866-018-1300-y (PMC6194671; doi:10.1186/s12866-018-1300-y)

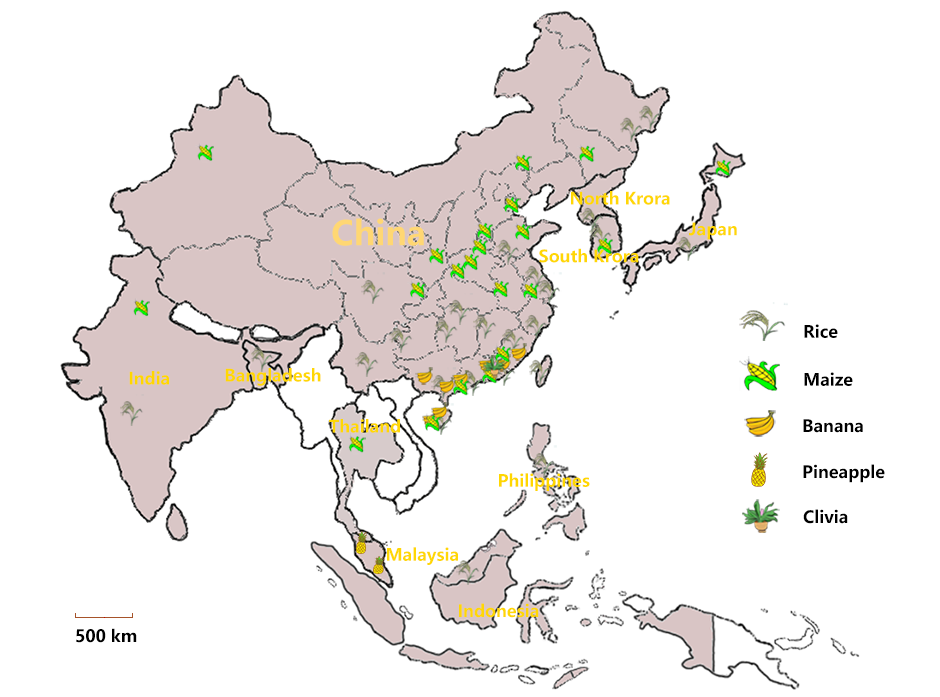

Supplement: Supplementary file 2 — Natural hosts and distribution of D. zeae strains in southeast Asia. The map was drawn using Photoshop CS6 software and host plant icons were added on the corresponding locations of the map. (TIF 2891 kb) [file 12866_2018_1300_MOESM2_ESM.tif]
