# Supplementary material for: Dickeya zeae strains isolated from rice, banana and clivia rot plants show great virulence differentials
Source: BMC Microbiol. 2018 Oct 18;18:136. doi: 10.1186/s12866-018-1300-y (PMC6194671; doi:10.1186/s12866-018-1300-y)
Supplement: Supplementary file 3 — The diseased symptoms of the tested strains on dicotyledonous and monocotyledonous hosts corresponding to Table 1. (PDF 6199 kb) [file 12866_2018_1300_MOESM3_ESM.pdf]

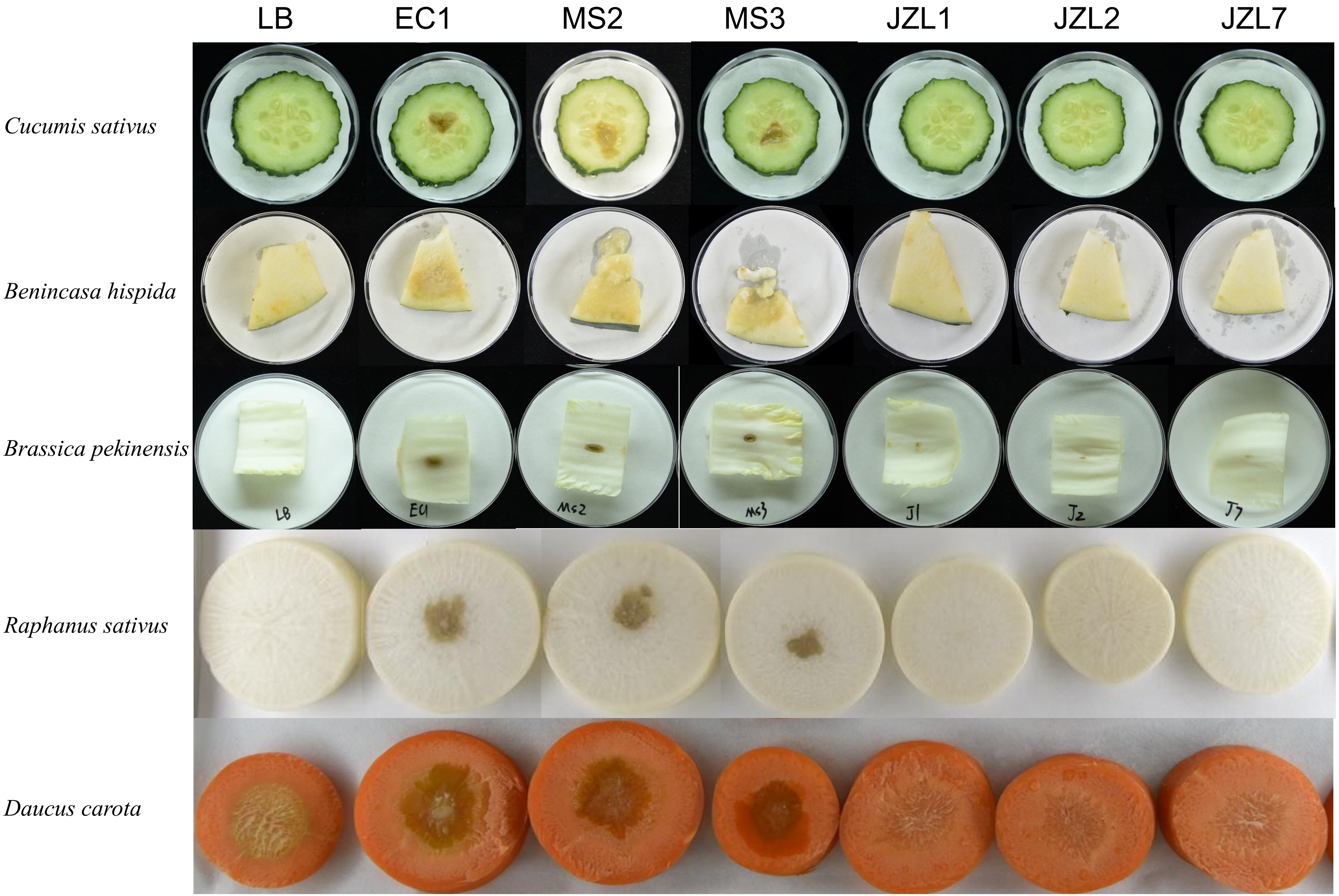
LB
EC1
MS2
MS3
JZL1
JZL2
JZL7
Cucumis sativus
Benincasa hispida
Brassica pekinensis
Raphanus sativus
Daucus carota

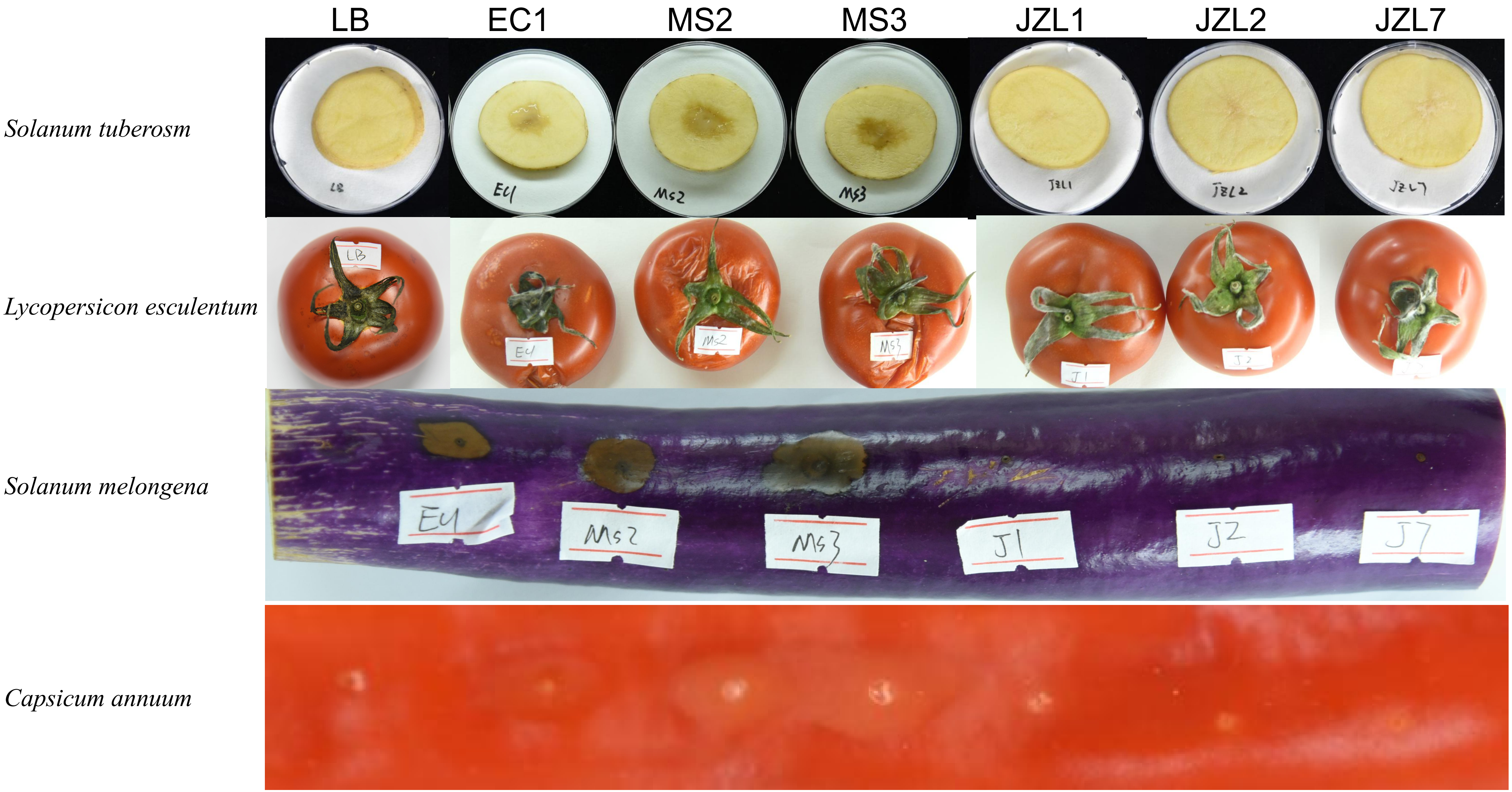
LB
EC1
MS2
MS3
JZL1
JZL2
JZL7
Solanum tuberosm
Lycopersicon esculentum
Solanum melongena
Capsicum annuum

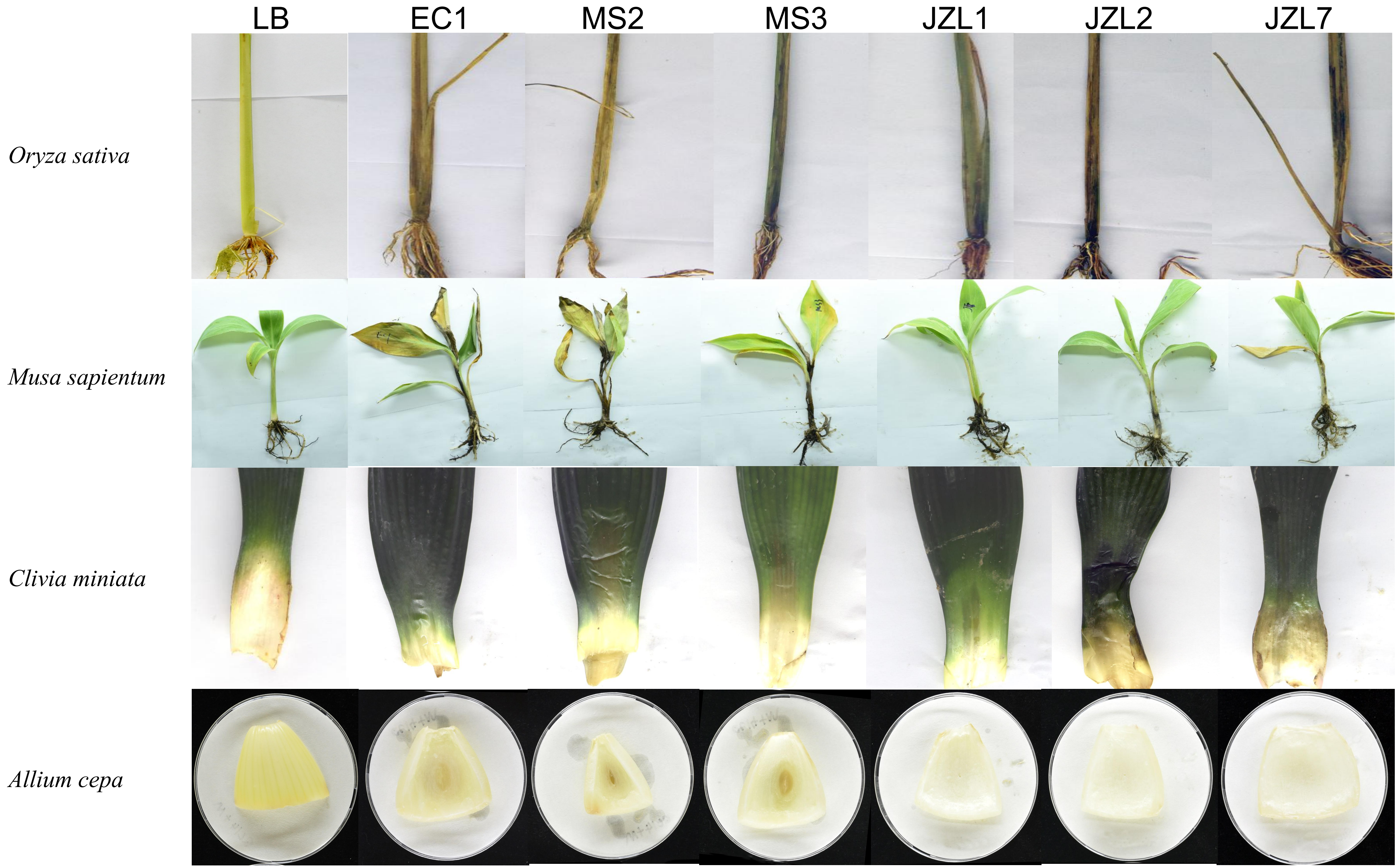

LB
EC1
MS2
MS3
JZL1
JZL2
JZL7
Oryza sativa
Musa sapientum
Clivia miniata
Allium cepa

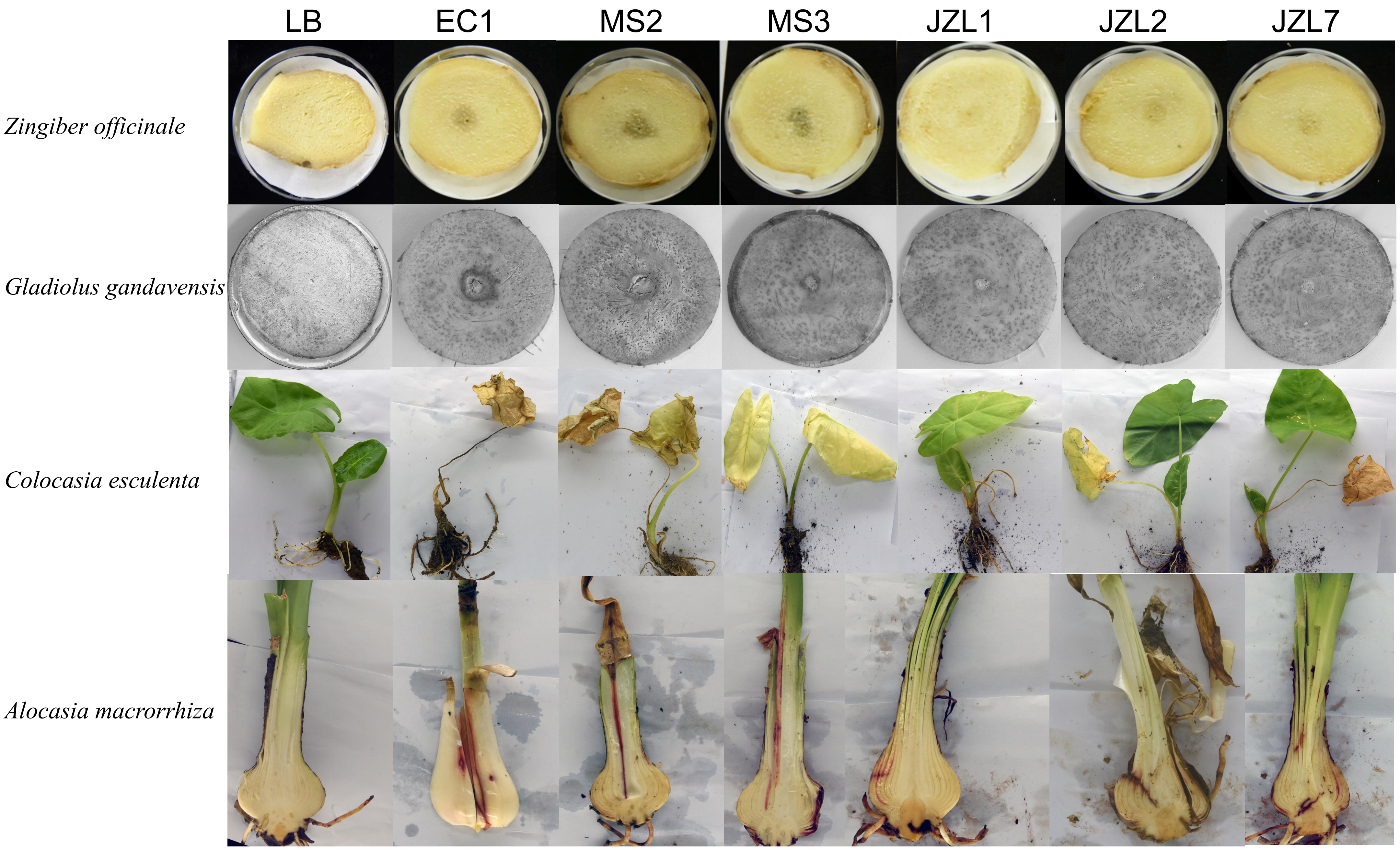
LB
EC1
MS2
MS3
JZL1
JZL2
JZL7
Zingiber officinale
Gladiolus gandavensis
Colocasia esculenta
Alocasia macrorrhiza
