# Supplementary material for: Dickeya zeae strains isolated from rice, banana and clivia rot plants show great virulence differentials
Source: BMC Microbiol. 2018 Oct 18;18:136. doi: 10.1186/s12866-018-1300-y (PMC6194671; doi:10.1186/s12866-018-1300-y)
Supplement: Supplementary file 4 — Bacterial strains used in this study. (DOC 36 kb) [file 12866_2018_1300_MOESM4_ESM.doc]

**Additional file 4**. Bacterial strains used in this study.

| **Strain** | **Description** | **Source or reference** |
| --- | --- | --- |
| ***Dickeya* spp.** | | |
| EC1 | Wild-type rice foot rot pathogen | [7] |
| 3937 | *D. dadantii* strain infects African violets | [11,38] |
| MS2 | Strain isolated from soft rot banana plant collected from Nansha District in Guangdong, China | This study |
| MS3 | Strain isolated from soft rot banana plant collected from Panyu District in Guangdong, China | This study |
| JZL1 | Strain isolated from soft rot *Clivia miniata* plants collected from Fangcun flower market in Guangzhou, China | This study |
| JZL2 | Strain isolated from soft rot *Clivia miniata* plants collected from Fangcun flower market in Guangzhou, China | This study |
| JZL7 | Strain isolated from soft rot *Clivia miniata* plants collected from Fangcun flower market in Guangzhou, China | This study |
| ***Escherichia coli*** | | |
| DH5α | *supE44△lacU169 (φ80lacZ△M15) hsdR17 recA1 endA1 gyrA96 thi-1 relA1 λpir* | Laboratory collection |
| OP50 | A food source for *C. elegans* | Laboratory collection |
