# Supplementary material for: Dickeya zeae strains isolated from rice, banana and clivia rot plants show great virulence differentials
Source: BMC Microbiol. 2018 Oct 18;18:136. doi: 10.1186/s12866-018-1300-y (PMC6194671; doi:10.1186/s12866-018-1300-y)
Supplement: Supplementary file 5 — Primers used in this study for detection of zeamines biosynthesis genes. (DOC 54 kb) [file 12866_2018_1300_MOESM5_ESM.doc]

**Additional file 5.** Primers used in this study for detection of zeamines biosynthesis genes.

| **Primer name** | **Primer sequence (5’-3’)** | **Amplicon** |
| --- | --- | --- |
| *zmsO-F* | GTTTTGAAGCGCAGGGTAGA | *zmsO* detection |
| zmsO-R | ACTGGCAGATGCTAGGTTGT |
| *zmsP-F* | TGAAGGGGGCATGGTTAGTA | *zmsP* detection |
| *zmsP-R* | GGGGCCAAACACTTCACTTA |
| *zmsQ-F* | ATCATTGGTCCTTCTGGCTC | *zmsQ* detection |
| *zmsQ-R* | CGTGCTCGATCAGACGATT |
| *zmsR-F* | GGTAAACGGGTCCACATAGC | *zmsR* detection |
| *zmsR-R* | CAGACAGTAGCGTGATGGTC |
| *zmsS-F* | TTGATGCACTTGGTCGTGTC | *zmsS* detection |
| *zmsS-R* | GTGTAACAAGCCAGCACGAA |
| *zmsA-F* | ATGACGATTTCTCCCACTGC | *zmsA* detection |
| *zmsA-R* | TCTCTATGCGTTTGTCTGCC |
| *zmsB-F* | ATGGTTGTCTGCGCTATCAG | *zmsB* detection |
| *zmsB-R* | ACAACGTCATAGGTAACGCC |
| *zmsC-F* | CGCAATTCACGAGTATGGGA | *zmsC* detection |
| *zmsC-R* | CGAGCATTTTCCATCGCATC |
| *zmsD-F* | AGCCAACAAGTCGCTGTTAT | *zmsD* detection |
| *zmsD-R* | CTCCGGTGAACCAATCATGT |
| *zmsE-F* | ATGTGAAAGGTACGCAGCAT | *zmsE* detection |
| *zmsE-R* | AACAGTGCCATCAAATCGGT |
| *zmsF-F* | TGTCGGGGTAGTGACATCAT | *zmsF* detection |
| *zmsF-R* | TTTCCCCCAGAGGATCTACC |
| *zmsG-F* | GCGATAGAGTTCCAGCAACA | *zmsG* detection |
| *zmsG-R* | GGCACCCGCTTTATTACTCA |
| *zmsI-F* | TTGGGTCATTGGTGGTGAAG | *zmsI* detection |
| *zmsI-R* | GCTGTTGTTCATCTCGTTGC |
| *zmsJ-F* | GCACCACAGATACCGTTCAT | *zmsJ* detection |
| *zmsJ-R* | AAAGCTATCATCACCCAGCG |
| *zmsK-F* | TGTGGATGAGGCGATTTGTC | *zmsK* detection |
| *zmsK-R* | GGAGCCATGAGCATTTCTGA |
| *zmsL-F* | TTATCGGGGCGAGTTTCTTG | *zmsL* detection |
| *zmsL-R* | GATGGGAAGTTCGGCATTCA |
| *zmsM-F* | GATGTGAAACGTGAGAGCGT | *zmsM* detection |
| *zmsM-R* | GCCAATACAGCAATACCCGA |
| *zmsN-F* | ATGATCGAGCCCAGGAAGTA | *zmsN* detection |
| *zmsN-R* | GTTGGCAGTGAAAATCCGTC |
